# Dactolisib outperforms Rapamycin in potentiating apoptosis induced by XPO1, Bcl‐2 and Mcl‐1 blockade in preclinical glioblastoma models

**DOI:** 10.1002/2211-5463.70357

**Published:** 2026-10-24

**Authors:** Kai Zhao, Leonie Meyer, Madita Braun, Katharina Otte, Celine Bitter, Maite Pawlik, Hartmann Raifer, Frederik Helmprobst, Axel Pagenstecher, Hans Urban, Michael C. Burger, Michael W. Ronellenfitsch, Joachim P. Steinbach, Jarmila Jedelská, Shamim Bagheri, Behrooz H. Yousefi, Pietro Di Fazio, Markus Luster, Andreas H. Mahnken, Andreas Neubauer, Andreas Burchert, Christopher Nimsky, Jörg W. Bartsch, Tillmann Rusch

**Affiliations:** ^1^ Department of Hematology, Oncology & Immunology Philipps‐University Marburg, UKGM Marburg Germany; ^2^ Department of Neurosurgery Philipps‐University Marburg, UKGM Marburg Germany; ^3^ FACS Core Facility Philipps‐University Marburg Germany; ^4^ Department of Neuropathology Philipps‐University Marburg, UKGM Marburg Germany; ^5^ Core Facility Mouse Pathology and Electron Microscopy Philipps‐University Marburg Germany; ^6^ University Cancer Center (UCT) Frankfurt‐Marburg Germany; ^7^ Dr. Senckenberg Institute of Neurooncology Goethe‐University Frankfurt Germany; ^8^ Department of Diagnostic and Interventional Radiology Philipps‐University Marburg, UKGM Marburg Germany; ^9^ Department of Nuclear Medicine Philipps‐University Marburg, UKGM Marburg Germany

**Keywords:** apoptosis resistance, BH3‐mimetics, dactolisib, glioblastoma, glioblastoma stem‐like cells, PI3K/mTOR dual inhibition

## Abstract

Glioblastoma resists therapy through interconnected survival programs. Combined XPO1, Bcl‐2, and Mcl‐1 inhibition (Eltanexor, Venetoclax, A1210477; EVA) enhances the efficacy of chemotherapy, yet resistance persists. We hypothesized that PI3K/AKT/mTOR (PAM) signaling drives adaptive escape. TCGA/GTEx analyses and immunohistochemistry indicated PAM pathway activation in glioblastoma, with AKT significantly overexpressed, whereas PIK3CA showed a clear trend towards higher expression, although it did not reach statistical significance. qPCR analysis showed that PAM‐component expression was highest in patient‐derived glioblastoma stem‐like cells (GSCs). In U87, U251, and GSCs, the dual PI3K/mTOR inhibitor Dactolisib combined with EVA and 5‐Azacytidine reduced viability and induced apoptosis more effectively than any of the Rapamycin combinations, and the difference in apoptosis was most pronounced in U87 and GSCs. Dactolisib monotherapy shifted the cell cycle towards G1 with a reduced S‐phase fraction. In CAM xenografts, [^18^F]FDG‐PET/CT showed decreased tracer uptake, and Caspase‐3 activation with PARP cleavage was detected only under EVA + Dactolisib treatment. Taken together, our results suggest that dual PI3K/mTOR inhibition with Dactolisib disables a critical adaptive survival axis, warranting further preclinical evaluation.

## Graphical abstract

The PAM pathway and its impact on cellular metabolism. Color‐coded key symbols represent Rapamycin (R, orange, targeting mTOR) and Dactolisib (D, green, targeting PI3K/mTOR), indicating their sites of action. Created with BioRenders.com.

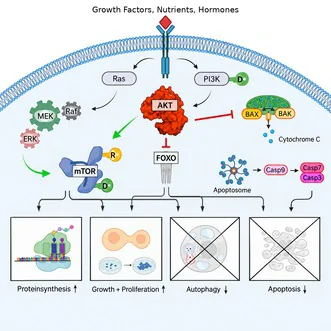

**Abbreviations**
%ID·mL^−1^
: percentage of the injected dose per milliliter
[18F]FDG: 2‐deoxy‐2‐[18F]fluoro‐D‐glucose
[18F]FET: O‐(2‐[18F]fluoroethyl)‐L‐tyrosine
4EBP: eukaryotic translation initiation factor 4E‐binding protein
5‐Aza: 5‐azacytidine
Ara‐C: cytarabine
ATCC: American Type Culture Collection
BBB: blood–brain barrier
BCA: bicinchoninic acid
bFGF: basic fibroblast growth factor
BME: basal medium eagle
CAM: chorioallantoic membrane
CpG: 5′‐cytosine–phosphate–guanine‐3′ dinucleotide
Ctrl: untreated control
D: dactolisib (in drug combination labels)
DICOM: digital imaging and communications in medicine
E: eltanexor (in drug combination labels)
ECACC: European Collection of Authenticated Cell Cultures
EVA + D: EVA plus Dactolisib
EVA + R: EVA plus Rapamycin
EVA: combination of Eltanexor, Venetoclax and A1210477
FOXO: Forkhead box O
Gabarapl1: GABA type A receptor‐associated protein‐like 1
GEPIA2: gene expression profiling interactive analysis, version 2
GFAP: glial fibrillary acidic protein
GSC: glioblastoma stem‐like Cell
GTEx: genotype‐tissue expression project
H&E: hematoxylin and eosin
HSD: honestly significant difference (Tukey's *post hoc* test)
ICLAC: International Cell Line Authentication Committee
MDM2: mouse double minute 2 homolog
MDSC: myeloid‐derived suppressor cell
MEM: minimum essential medium
MTX: methotrexate
NF1: neurofibromin 1
p14ARF (ARF): alternative reading frame protein p14
PAM: PI3K/AKT/mTOR (signaling pathway)
PARP: Poly(ADP‐Ribose) polymerase
PDGFRA: platelet‐derived Growth Factor Receptor Alpha
PFA: paraformaldehyde
PIK3CA: phosphatidylinositol‐4,5‐bisphosphate 3‐kinase catalytic subunit alpha
PTEN: phosphatase and tensin homolog
R: Rapamycin (in drug combination labels)
RPLP0: ribosomal protein lateral stalk subunit P0
RTK: receptor tyrosine kinase
TBS: tris‐buffered saline
TCGA: The Cancer Genome Atlas
TERT: telomerase reverse transcriptase
TMZ: Temozolomide
TTF: tumor‐treating fields
V: Venetoclax (in drug combination labels)
XPO1: Exportin 1

Glioblastoma is the most common and aggressive primary brain tumor in adults, with a poor prognosis despite intensive multimodal treatment including surgery, radiochemotherapy, and tumor‐treating fields (TTF) [1, 2]. Median overall survival under standard therapy is commonly reported as 14–16 months, whereas recent real‐world data, applying the 2021 WHO classification, suggest an even shorter glioblastoma‐specific survival of approximately 8 months [3, 4, 5]. Although recent clinical trials show promise [6, 7, 8], a true breakthrough remains elusive due to the remarkable ability of glioblastoma cells to rapidly develop therapeutic resistance [9]. Understanding and targeting these resistance mechanisms is therefore crucial for developing novel therapies.

One major driver of therapy resistance is the PI3K/AKT/mTOR (PAM) signaling cascade, which is dysregulated in nearly 90% of glioblastoma cases, where it promotes proliferation, survival, and metastasis [10] (Graphical Abstract). Under physiological conditions, the PAM pathway regulates protein synthesis, energy metabolism, neuronal plasticity, and autophagy in response to nutrient and growth factor signals [11, 12]. In cancer, however, constitutive pathway activation drives tumor progression through enhanced metabolism, proliferation, apoptosis resistance, motility, and angiogenesis, making it a compelling therapeutic target [13, 14].

Mechanistically, receptor tyrosine kinase signaling activates PI3K, which converts PIP2 into PIP3 [15, 16], leading to activation of AKT isoforms (AKT1–3) [17, 18, 19]. Activated AKT in turn regulates multiple downstream effectors that collectively suppress apoptosis and promote cell survival [20]: it stimulates mTORC1 by phosphorylating TSC2, thereby enhancing protein and lipid synthesis, cell growth, and proliferation while inhibiting autophagy [21, 22, 23]. Notably, mTORC1 can also be activated independently of AKT through the Ras–Raf–MEK–ERK cascade [24, 25] (Graphical Abstract). Beyond mTOR, AKT silences FOXO transcription factors through phosphorylation‐induced nuclear exclusion and degradation, which suppresses pro‐apoptotic genes such as Bim and FasL and cell cycle inhibitors including CDKN1B [26, 27, 28]. Under stress conditions, FOXOs conversely induce autophagy via LC3, Atg12, and Gabarapl1 [29] (Graphical Abstract). Additionally, AKT directly phosphorylates the pro‐apoptotic protein BAX, preventing its mitochondrial translocation and blocking BAX–BAK oligomerization, a key step in cytochrome c release and intrinsic apoptosis [30, 31] (Graphical Abstract). Together, these mechanisms establish the PAM pathway as a central hub of apoptosis evasion in glioblastoma.

Efforts to therapeutically target this pathway have yielded mixed results. Rapamycin, an mTORC1 inhibitor, reduces glioblastoma cell proliferation and induces autophagy [32], yet single‐agent clinical efficacy has been limited, partly due to incomplete pathway blockade and paradoxical feedback activation [33]. Dactolisib, a dual PI3K/mTOR inhibitor, offers broader pathway suppression and induces apoptosis in preclinical glioblastoma models [34]. Although early clinical studies revealed dose‐limiting toxicity, they confirmed Dactolisib's biological activity, warranting further development in rational combination strategies [35].

Previous work from our lab demonstrated the *in vitro* efficacy of combining the XPO1 inhibitor Eltanexor, the Bcl‐2 inhibitor Venetoclax, and the Mcl‐1 inhibitor A1210477, hereafter referred to as EVA, together with one of the chemotherapeutics Temozolomide (TMZ), Methotrexate (MTX), or Cytarabine (Ara‐C) [36, 37]. We showed that MTX and Ara‐C are 150–10 000‐fold more potent than TMZ at inducing apoptosis, and that EVA overcame compensatory upregulation of its targets triggered by chemotherapy, producing strong synergistic reductions in viability with robust caspase‐3/7 activation and G1 arrest in glioblastoma cell lines and patient‐derived GSCs. However, residual resistance persisted, raising the question of whether additional survival pathways sustain glioblastoma cells under EVA pressure. This study therefore investigates whether PAM signaling serves as an adaptive escape route under EVA therapy, and whether combining EVA with Rapamycin or Dactolisib can overcome this resistance.

We additionally included 5‐Azacytidine (5‐Aza), a DNA‐methyltransferase inhibitor with demonstrated direct antitumor activity in glioblastoma models that is independent of DNA‐damage‐induced apoptosis [38]. 5‐Aza is moreover one of the few agents for which intrathecal administration in recurrent high‐grade glioma has already entered clinical testing, in a phase 1 trial combining intrathecal 5‐Aza with Nivolumab [39], which makes it particularly relevant for the locoregional delivery concept proposed here.

The rationale for the resulting combinations is not empirical but follows a defined sequence of escape mechanisms, each step of which was derived experimentally from the preceding one. It thereby addresses the central cause of treatment failure in glioblastoma, namely the repeated adaptation of the tumor to existing therapeutic approaches and the subsequent emergence of therapy‐ and apoptosis‐resistant recurrences: (a) chemotherapeutic agents (TMZ, MTX, Ara‐C, 5‐Aza) induce apoptotic signaling; (b) this induction is counteracted by compensatory upregulation of Bcl‐2, Mcl‐1, and XPO1, which are blocked by EVA [36, 37]; (c) EVA in turn is accompanied by transcriptional induction of PAM pathway components, identifying this axis as the next escape route and forming the hypothesis tested here; (d) Rapamycin and Dactolisib were selected as the two clinically available inhibitors that differ precisely in the completeness of PAM blockade (mTORC1 only versus PI3K together with mTORC1/2), which makes them the appropriate pair to test whether the completeness of blockade determines the outcome. Throughout this study, all agents are used at sublethal single‐agent concentrations, that is, at doses that produce little or no effect on their own. The rationale of the approach is therefore not additive cytotoxicity at full dose, but the sequential removal of apoptotic brakes at low dose.

## Materials and methods

### Clinical specimens

All tumor samples were histopathologically confirmed by a board‐certified neuropathologist (A.P.) at University Hospital Marburg. This study was approved by the Ethics Committee of Philipps University Marburg (file number 185/11). The experiments were undertaken with the understanding and written consent of each individual subject. The study methodologies conformed to the standards set by the Declaration of Helsinki.

### Gene expression and immunohistochemistry

Gene expression levels in normal brain and glioblastoma tissues were analyzed using the ‘Expression Analysis – Box Plots’ module on GEPIA2 (http://gepia2.cancer‐pku.cn/#analysis; accessed August 8, 2024), based on GTEx and TCGA datasets, to illustrate differential expression of mTOR, AKT, and PIK3CA. Immunohistochemical staining for AKT2 and PIK3CA in paraffin‐embedded sections from representative glioblastoma and normal brain samples was retrieved from the Human Protein Atlas (https://www.proteinatlas.org; accessed April 8, 2025).

### Chemotherapeutic agents and inhibitors

Temozolomide (S1237) and Eltanexor (S8397) were purchased from Selleck Chemicals (Houston, TX, USA). Methotrexate (MTX, HY‐14519), Cytarabine (Ara‐C, HY‐13605), Venetoclax (HY‐15531), A1210477 (HY‐12468), Rapamycin (HY‐10219), and Dactolisib (HY‐50673) were purchased from MedChemExpress (Monmouth Junction, NJ, USA).

### Cell isolation and cell culture

U87 (obtained from ATCC, HTB‐14; RRID: CVCL_0022) and U251 (obtained from ECACC, Cat‐Nr.: #09063001; RRID: CVCL_0021) human glioblastoma cell lines were cultured in DMEM (41965039, Gibco) supplemented with 10% FBS (FBS‐16A, Capricorn), 1% sodium pyruvate (12539059, Gibco, Eggenstein‐Leopoldshafen, Germany), 1% penicillin–streptomycin (FS‐B, Capricorn Scientific, Dreihausen, Germany), and 1% NEAA (12084947, Gibco). Both cell lines were delivered with a recent authentication by the provider. Both lines were selected for a molecular background that bears directly on the question addressed in this study. The established human glioblastoma lines U87MG (RRID:CVCL_0022; hereafter U87) and U251MG (RRID:CVCL_0021; hereafter U251) were used. Both lines were selected because they combine constitutive PAM pathway activation with divergent TP53 status. Both are PTEN‐deficient, U87 carrying a homozygous splice‐donor mutation (PTEN c.209+1G>T) and U251 a frameshift mutation (PTEN p.Glu242Valfs*15) [40], consistent with the constitutive PI3K/AKT/mTOR signaling reported for both lines [41]. U87 is TP53 wild‐type, whereas U251 carries the homozygous hotspot mutation p.Arg273His [40]; in both lines the p53 axis is additionally affected by a homozygous CDKN2A/p14ARF deletion, which also removes the ARF–MDM2–p53 arm [42]. Both lines are IDH1 wild‐type and carry the TERT promoter mutation C228T [40], and neither expresses detectable MGMT mRNA or protein, in line with the methylation reported for the clinically relevant CpG sites of the MGMT promoter and exon 1 [43]. U87 additionally carries a heterozygous NF1 frameshift mutation (p.Phe1247Ilefs*18) [40]; neither line carries an EGFR or PDGFRA amplification [41].

No transcriptional or DNA‐methylation‐based subtype assignment (RTK I/RTK II/mesenchymal and the corresponding TCGA expression subtypes) was performed in the present study; the two lines are characterized here by their subtype‐defining alterations. Neither line carries the EGFR amplification characteristic of the classical/RTK II group, whereas the combination of a truncating NF1 mutation with PTEN loss in U87 corresponds to the alteration pattern associated with the mesenchymal subtype of the TCGA classification [44]. Published expression‐based assignments for these lines are not concordant: both have been assigned to the proneural group on the basis of their genomic alterations [41], whereas transcriptome analysis has ascribed a neural fingerprint to U87 [45]. Prolonged serum culture shifts glioblastoma cells away from the expression profile of the parental tumor [46].

With respect to provenance, the ATCC U87 stock is registered by ICLAC as a misidentified line: it is a bona fide human glioblastoma line, but not the original U87MG established in Uppsala in 1968, and its donor is unknown [47]. Long‐term U251 cultures undergo genetic drift, so that sublines of different provenance may differ genetically and phenotypically [48].

Glioblastoma stem‐like cells (GSCs) were isolated as previously described [49] by mechanical and enzymatic dissociation (accutase, 00‐4555‐56, ThermoFisher Scientific, Darmstadt, Germany; 30 min at 37 °C) of glioma tissue, followed by filtration (40 μm; 542040, Greiner Scientific, Frickenhausen, Germany) and red blood cell lysis (R7757, MilliporeSigma, Taufkirchen, Germany). Cells were plated on laminin‐coated (20 μg·mL^−1^; 11243217001, Roche, Mannheim, Germany) six‐well plates in PBS and cultured under sphere‐forming conditions. GSCs were maintained in DMEM/F12 (DMEM‐12‐A, Capricorn) supplemented with 2% B27 (117504044, Gibco), 1% amphotericin (152290026, Gibco), 0.5% HEPES (H0887, Sigma), 0.1% gentamycin (A2712, Biochrom AG, Berlin, Germany), and 20 ng/mL each of EGF (100‐18B, Peprotech/Gibco, Hamburg, Germany) and bFGF (315‐09, Peprotech). All cultures were incubated at 37 °C with 5% CO_2_ and routinely tested for mycoplasma and authentication. The cultures were not subjected to CD133‐based sorting and therefore represent serum‐free sphere cultures enriched for cells with a stem‐like phenotype rather than a purified CD133‐positive fraction; they are referred to as glioblastoma stem‐like cells throughout. The stem‐like phenotype was characterized functionally rather than by a single surface marker [49]: exposure to FCS abolished sphere growth and led to downregulation of CD133 with induction of GFAP; side population analysis identified a Hoechst‐33342‐effluxing fraction that was abolished by verapamil; the cells were significantly more invasive than U87 in the Matrigel transwell assay; and after stereotactic intracranial injection into nude mice, they formed diffusely infiltrating tumors spreading along the corpus callosum, in contrast to the circumscribed masses formed by U87. The donor tumor was a histologically confirmed glioblastoma with a methylated MGMT promoter and no detectable IDH1 R132H mutation [49]; as for the established lines, a transcriptional or DNA‐methylation‐based subtype (RTK‐associated versus mesenchymal) was not determined.

### Cell viability assay

Cell viability of U87, U251, and GSCs was measured using the CellTiter‐Glo® 3D Assay (G7571, Promega GmbH, Walldorf, Germany). U87/U251 cells (4 × 10^3^/well) and GSCs (1 × 10^4^/well) were seeded in 96‐well plates and incubated overnight. Drug‐treated cells were assessed after 3 days (U87/U251) or 10 days (GSCs). For detection, 50 μL reagent was added per well, followed by 15 min shaking and 15 min incubation in the dark. Luminescence was read with a FLUOstar OPTIMA Microplate Reader (BMG LABTECH).

### Apoptosis assay

Apoptosis rates were determined using luminogenic caspase‐3/7 assays: Caspase‐Glo® 3/7 (G8090, Promega) for U87/U251 cells and Caspase‐Glo® 3/7 3D (G8981, Promega) for GSCs. U87/U251 cells (4 × 10^3^/well) and GSCs (1 × 10^4^/well) were seeded in 96‐well plates and incubated overnight. After drug treatment, apoptosis was assessed at 24 h (U87/U251) and 48 h (GSCs). For detection, 20 μL reagent was added per well, shaken (30 s), incubated for 60 min at room temperature in the dark, and luminescence measured using a FLUOstar OPTIMA Microplate Reader (BMG LABTECH, Ortenberg, Germany).

### RNA isolation and Real‐time qPCR

Cells were treated with the indicated drugs prior to RNA extraction using QIAzol (79306, Qiagen, Hilden, Germany). Only samples with OD260/280 between 1.8 and 2.1 were used. Two micrograms of RNA were reverse transcribed using RNA to cDNA EcoDry Premix (Takara Bio Europe SAS, Saint‐Germain‐en‐Laye, France). qPCR was performed with 10 μL SYBR Green/Rox Master Mix (Bio‐Rad Laboratories GmbH, Feldkirchen‐Heimstetten, Germany), 2 μL primers, 6 μL water, and 2 μL cDNA. Cycling conditions: 95 °C for 10 min, then 40 cycles of 95 °C for 15 s and 60 °C for 1 min. Primers for *mTOR*, *AKT2*, *PIK3CA*, *Bcl‐2*, *Mcl‐1*, and *XPO1* (QuantiTect®, Qiagen); *RPLP0* served as reference gene. Relative expression was calculated via the $2^{–\Delta \Delta C_{T}}$ method and visualized as heat maps.

### Protein isolation and western blot analysis

Cells and tumor tissues were treated with the indicated drugs before protein extraction. After three PBS washes, proteins were extracted from homogenized tissue (IKA Ultra‐Turrax) and cell pellets using RIPA buffer with phenanthroline, protease (A32955), and phosphatase inhibitors (A32957, Thermo Fisher Scientific). Protein concentrations were measured via BCA Assay (23250, Thermo Fisher Scientific). 20 μg protein/sample was mixed with reducing buffer (B0009, Invitrogen/Thermo Fisher Scientific, Darmstadt, Germany) and Laemmli, boiled for 5 min, separated on 12.5% SDS/PAGE, and transferred to nitrocellulose membranes (A29591442, GE Healthcare, Düsseldorf, Germany). Membranes were blocked with 5% milk (T145.3, Carl Roth GmbH & Co.KG, Karlsruhe, Germany) for 1 h at room temperature, incubated overnight at 4 °C with primary antibodies, then for 1 h with secondary antibodies (Table S1). After final washes, detection was performed using ChemiDoc MP Imaging System (Bio‐Rad). Full, uncropped membranes including molecular weight markers are provided for all blots shown in this study in the supplementary material, in which the region reproduced in each figure panel is outlined.

### Cell cycle arrest analysis

Cell cycle analysis was performed using propidium iodide (PI; P4170, Sigma). U87 and U251 cells (1 × 10^6^) were seeded, incubated overnight, and treated with the indicated drugs for 24 h. Cells were washed with PBS, fixed in 80% cold ethanol for ≥ 12 h, then resuspended in 1 mL PI buffer containing 0.1% Triton X‐100 (T8787, Sigma) and 1 mg RNase A (EN0531, Thermo Fisher Scientific). After 30 min incubation at room temperature in the dark, cells were washed and resuspended in 300 μL PBS for flow cytometry. Three independent experiments were performed. For each experiment, the percentages of cells in G1, S, and G2/M phase were normalized to their sum, and the resulting distributions are given as mean ± SD of the three experiments.

### Brain slice culture and immunofluorescence staining

Organotypic cerebellar slice cultures were generated from 8 to 9 day‐old mice using a modified interface technique [50, 51]. Six‐well plates were pre‐filled with 1 mL medium composed of 25% BME, 25% heat‐inactivated horse serum, 1.3% glucose, 1% GlutaMax, all in MEM. Mice were deeply anesthetized with isoflurane and killed by cervical dislocation; the cerebella were removed immediately thereafter, and 400 μm slices were prepared using a McIlwain™ Tissue Chopper. Slices were placed in cold MEM, separated under a microscope, and transferred to Millicell® inserts (0.4 μm pores), three per membrane. Cultures were kept at 37 °C and 5% CO_2_, with medium changed the next day and every other day thereafter.

On day 8, slices were treated for 48 h with Venetoclax (10 μm), A1210477 (10 μm), Eltanexor (1 μm), 5‐Azacytidine (10 μm), Rapamycin (1 μm), or Dactolisib (100 nm). Staurosporine (50 μm, 12 h) served as a positive control; DMSO was used as vehicle. Slices were incubated with 2 μm PI for 5–8 h, then fixed in 4% PFA overnight at 4 °C. After TBS and PBS washes, slices were blocked in BSA/Triton/NaN_3_ overnight and incubated with anti‐active caspase‐3 antibody (AF835, 1:250) for 72 h. Following PBS washes, slices were stained with DyLight® 488‐conjugated secondary antibody (1:1000) for 72 h, counterstained with Hoechst, and imaged by confocal microscopy (Leica SP8i, Leica Microsystems, Wetzlar, Germany).

All experimental procedures involving mice were performed in accordance with the German Animal Protection Act (“Tierschutzgesetz”) and approved by the local authorities (Regierungspräsidium Gießen), file number G51/2021.

### Chorioallantoic membrane *in vivo* chicken embryo xenograft model

*In vivo* CAM assays were performed as previously described [52]. Fertilized chicken eggs (Brormann GmbH & Co.KG, Rheda‐Wiedenbrück, Germany) were equilibrated at room temperature for 2 h, disinfected with 70% ethanol, and incubated at 37.8 °C with 60–70% humidity (Easy 150, J. Hemel). On embryonic day 3, eggshell windows (~ 3 cm) were opened under sterile conditions, membranes removed, and viable embryos identified. Windows were resealed and eggs returned for incubation until day 8.

On day 8, 1 × 10^6^ U87 cells in 30 μL DMEM +15 μL Matrigel were applied to the CAM within sterile silicone rings. After 48 h, tumors were topically treated twice daily (days 11–13) with Eltanexor (100 nm), Venetoclax (1 μm), A1210477 (1 μm), Rapamycin (10 μm), or Dactolisib (250 nm). On day 14, tumors and adjacent CAM tissue were excised. Each tumor was halved: one half fixed in 4% PFA, paraffin‐embedded, sectioned (5 μm), H&E‐stained, and immunostained for Ki‐67 (mouse monoclonal anti‐human, Clone MIB‐1, Dako A/S, Glostrup, Denmark); the antibody is human‐specific and does not cross‐react with chicken Ki‐67. The other half was processed for protein extraction and western blot analysis (see section Protein isolation and western blot analysis).

### [ ^18^F]FDG injection and PET/CT acquisition

Imaging was performed using a Mediso nanoScan PET/CT system (Mediso Ltd., Budapest, Hungary), combining high‐resolution PET for functional imaging with CT for anatomical reference and attenuation correction, as previously described for CAM xenograft imaging [53]. [^18^F]FDG was used in preference to the amino‐acid tracer [^18^F]FET, which is the clinical standard in neuro‐oncology, for three reasons. The principal advantage of amino‐acid tracers in patients is that they avoid the high physiological glucose uptake of normal cortex, against which a tumor signal is difficult to resolve; in the CAM model the xenograft grows on the extraembryonic membrane without adjacent brain parenchyma, so that this background problem does not arise. Conversely, glycolytic activity is the functionally relevant endpoint here, because the PAM pathway directly controls glucose uptake and glycolysis, so that suppression of [^18^F]FDG uptake is a direct pharmacodynamic readout of the intervention under study rather than a surrogate for tumor mass. Finally, the routine availability of [^18^F]FDG in the local radiopharmacy allowed the required same‐day scheduling.

Radiotracer activity was measured in syringes immediately before and after injection using a VDC‐405 dose calibrator (Veenstra Instruments, Joure, The Netherlands). Tumor‐xenograft‐bearing chick embryos were injected with 5.8 ± 2.7 MBq [^18^F]FDG in a volume of up to 100 μL into a peripheral vein of the chorioallantoic membrane using a 33‐gauge needle (Mesoram, PromaMedical GmbH, Munich, Germany), as previously established [53]. Any bleeding at the injection site was controlled by gentle compression with a cotton swab. Embryos exhibiting blood perfusion across the chorioallantoic membrane or in proximity to tumor areas were excluded from analysis to avoid confounding of imaging data. Following injection, embryos were incubated for approximately 15 min to allow for adequate [^18^F]FDG distribution and tumor uptake.

Image acquisition was initiated with a CT scan in the transverse plane for morphological assessment and field‐of‐view determination (approximately 10 min), followed by a 10‐min static PET acquisition. PET/CT data were reconstructed using the Tera‐Tomo 3D iterative reconstruction algorithm (6 subsets, 8 iterations) [54]. Following imaging, embryos were sacrificed and tumors were excised for downstream analyses.

### PET/CT image analysis and quantification

Reconstructed DICOM data were imported into pmod software (version 4.305; PMOD Technologies LLC, Zurich, Switzerland) for image co‐registration and quantitative analysis. PET and CT datasets were co‐registered using rigid transformation. Two volumes of interest (VOIs) were manually delineated on the co‐registered images: one encompassing the tumor xenograft as the target region, and one encompassing the whole egg as reference. Radioactivity concentrations were automatically decay‐corrected by the nanoScan PET/CT acquisition system and expressed as percentage of the injected dose per milliliter (%ID·mL^−1^). Tumor‐to‐whole‐egg uptake ratios were subsequently calculated. The tumor‐to‐whole‐egg uptake ratio was selected as the primary quantitative endpoint because it normalizes for inter‐individual variability in [^18^F]FDG injection efficiency, which showed a coefficient of variation of approximately 47% across experiments. Statistical comparisons between treatment groups were performed using one‐way ANOVA followed by Tukey HSD *post hoc* test, with *n* = 3 eggs per treatment group.

### Statistics

All data are presented as mean ± standard deviation (SD) or standard error of the mean (SEM), as indicated. Statistical analyses were performed using graphpad prism (GraphPad Software Inc., San Diego, CA, USA). Differences were considered statistically significant at *P* < 0.05. Non‐significant results are indicated as ‘ns’ (*P* > 0.05). For comparisons involving multiple groups, one‐way analysis of variance (ANOVA) was applied with *post hoc* testing (Tukey HSD). The relationship between relative caspase‐3/7 activity and relative cell viability was assessed for each cell line by Spearman rank correlation. One data point per treatment group was used, defined as the mean of the nine values obtained in three independent experiments in triplicate; the untreated control was excluded because normalization fixes it at 1.00 for both variables, leaving *n* = 17 treatment groups per cell line. Caspase‐to‐viability ratios were calculated as the mean relative caspase‐3/7 activity of a treatment group divided by its mean relative cell viability.

## Results

### Expression levels of mTOR, AKT, and PIK3CA in glioblastoma tissue and cells

To assess *mTOR*, *AKT*, and *PIK3CA* expression in glioblastoma, data from TCGA and GTEx were analyzed. Of the three effectors analyzed, only AKT was significantly upregulated in glioblastoma relative to normal brain tissue (*P* < 0.05), whereas PIK3CA showed a clear trend towards higher expression that did not reach the applied significance threshold, and mTOR showed no relevant difference (Fig. 1A–C). Based on TCGA data, AKT2 and PIK3CA protein expression was further examined by immunohistochemistry (Fig. 1D–G). qPCR analysis of mTOR, AKT2, and PIK3CA mRNA levels in U87, U251, and patient‐derived GSCs revealed markedly higher expression in GSCs (Fig. 1H–J). PIK3CA was also significantly elevated in U251 cells compared to the other lines.

**Fig. 1:**
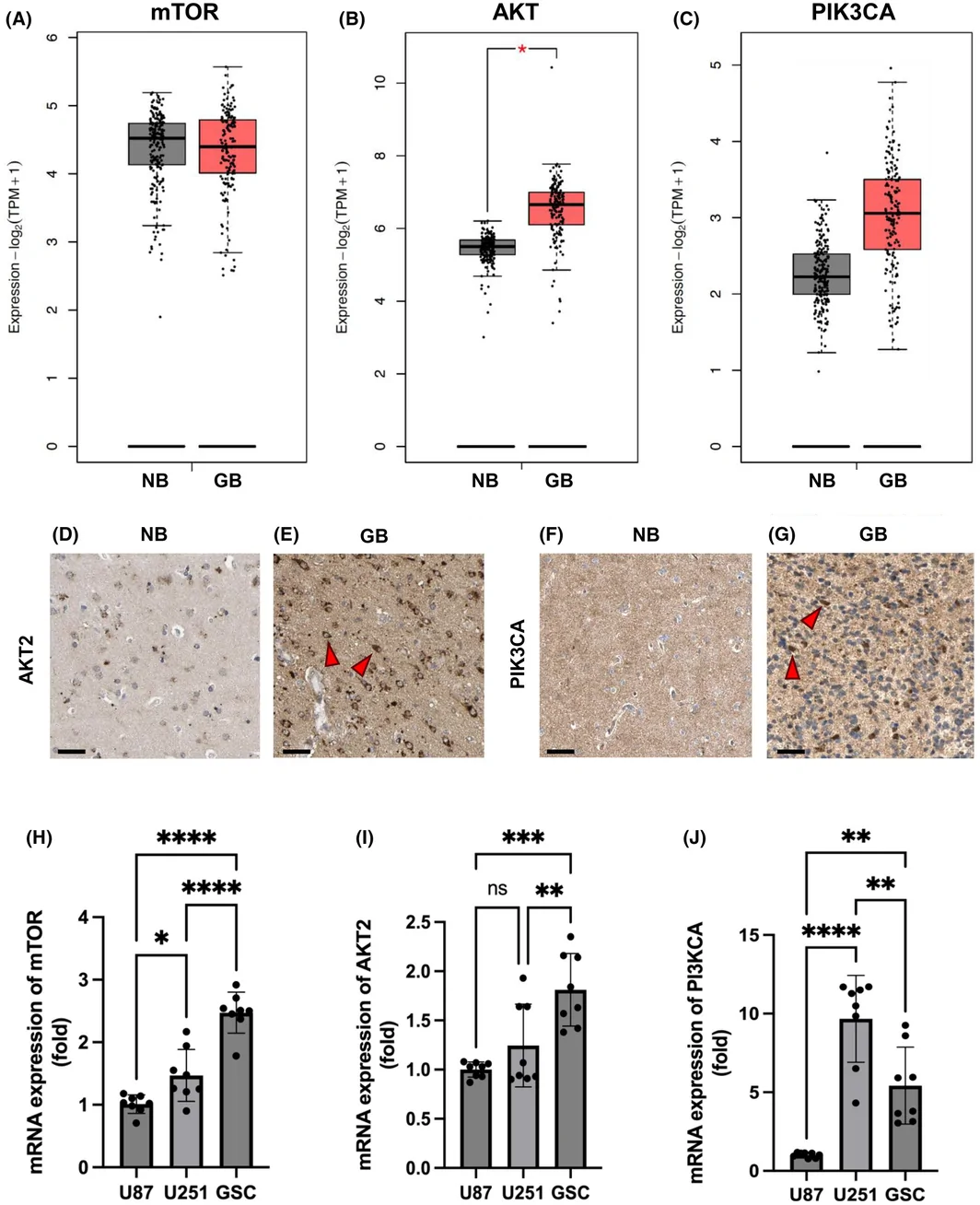
mRNA Expression and Immunohistochemistry of PAM pathway genes mTOR, AKT, and PIK3CA in glioblastoma compared to normal brain tissue. (A–C) Levels of mTOR, AKT, and PIK3CA in glioblastoma tumor (*n* = 163) compared to control brain tissue (*n* = 207), analyzed from TCGA and GTEx databases; *P < 0.05. (D–G) Immunohistochemistry of AKT2 (D, E) and PIK3CA (F, G) in paraffin sections from representative glioblastoma patients (E, G) and normal brain cortex (D, F), sourced from the Human Protein Atlas (https://www.proteinatlas.org). Positive staining is predominantly observed in tumor cells (red arrowheads), localized to the cytoplasm (AKT2) or nucleus (PIK3CA). Scale bars indicate 50 μm. (H–J) Relative mRNA expression levels of mTOR (H), AKT2 (I), and PIK3CA (J) in glioblastoma cell lines U87, U251, and a GSC‐line. Expression values are normalized to U87 (=1) and based on three independent experiments in triplicates. Data is presented as mean ± SD. Statistical significance was assessed using one‐way ANOVA. *P < 0.05, **P < 0.01, ***P < 0.001, ****P < 0.0001, ns = not significant.

### Cytotoxicity of Rapamycin, Dactolisib, and 5‐Aza in U87 cells, U251 cells, and GSCs

We evaluated the cytotoxicity of Rapamycin (mTOR), Dactolisib (mTOR/PI3K), and 5‐Azacytidine (5‐Aza) in U87, U251, and patient‐derived GSCs. Dactolisib showed IC_50_ values < 300 nm in all models (Table 1). Rapamycin was markedly less potent: 20.4 μm in U87 (vs. 228.1 nm Dactolisib), 1.2 μm in U251 (vs. 243.8 nm), and 315.4 nm in GSCs (vs. 104.7 nm). 5‐Aza displayed comparable IC_50_ values (~ 10 μm) across all cell types (Fig. S1).

**Table 1:** IC_50_ values for Rapamycin and Dactolisib in glioblastoma cell lines (U87, U251) and a patient‐derived glioblastoma stem‐like cell population (GSC). Cell viability was assessed using the CellTiter‐Glo® assay, and IC_50_ values were calculated by non‐linear regression with least‐squares fit. Data are based on three independent experiments.

Cells	IC_50_ (nm)	
	Rapamycin	Dactolisib
U87	20.404	228
U251	1.355	244
GSCs	327	105

### Cell viability and caspase activity resulting from combinatorial treatment with chemotherapeutic drugs (TMZ, MTX, Ara‐C, 5‐Aza) and Rapamycin or Dactolisib in glioblastoma cell lines and GSCs

As shown previously, TMZ, MTX, and Ara‐C induced cell death in glioblastoma cell lines and GSCs. Each chemotherapeutic agent (TMZ, MTX, Ara‐C or 5‐Aza) was combined pairwise with either Rapamycin or Dactolisib in U87, U251, and GSCs; Fig. 2 therefore shows two‐drug combinations only. Viability and apoptosis were measured via CellTiter‐Glo® and Caspase‐3/7 assays (Fig. 2A–F). In these pairwise combinations, Dactolisib reduced viability more effectively than Rapamycin in individual comparisons; the difference was statistically detectable but modest in magnitude. Notably, 5‐Aza in combination with Dactolisib strongly enhanced caspase activity, with a similar effect also observed when combined with Rapamycin in U87 cells. The full combination of chemotherapeutics, EVA and either Rapamycin or Dactolisib is addressed below.

**Fig. 2:**
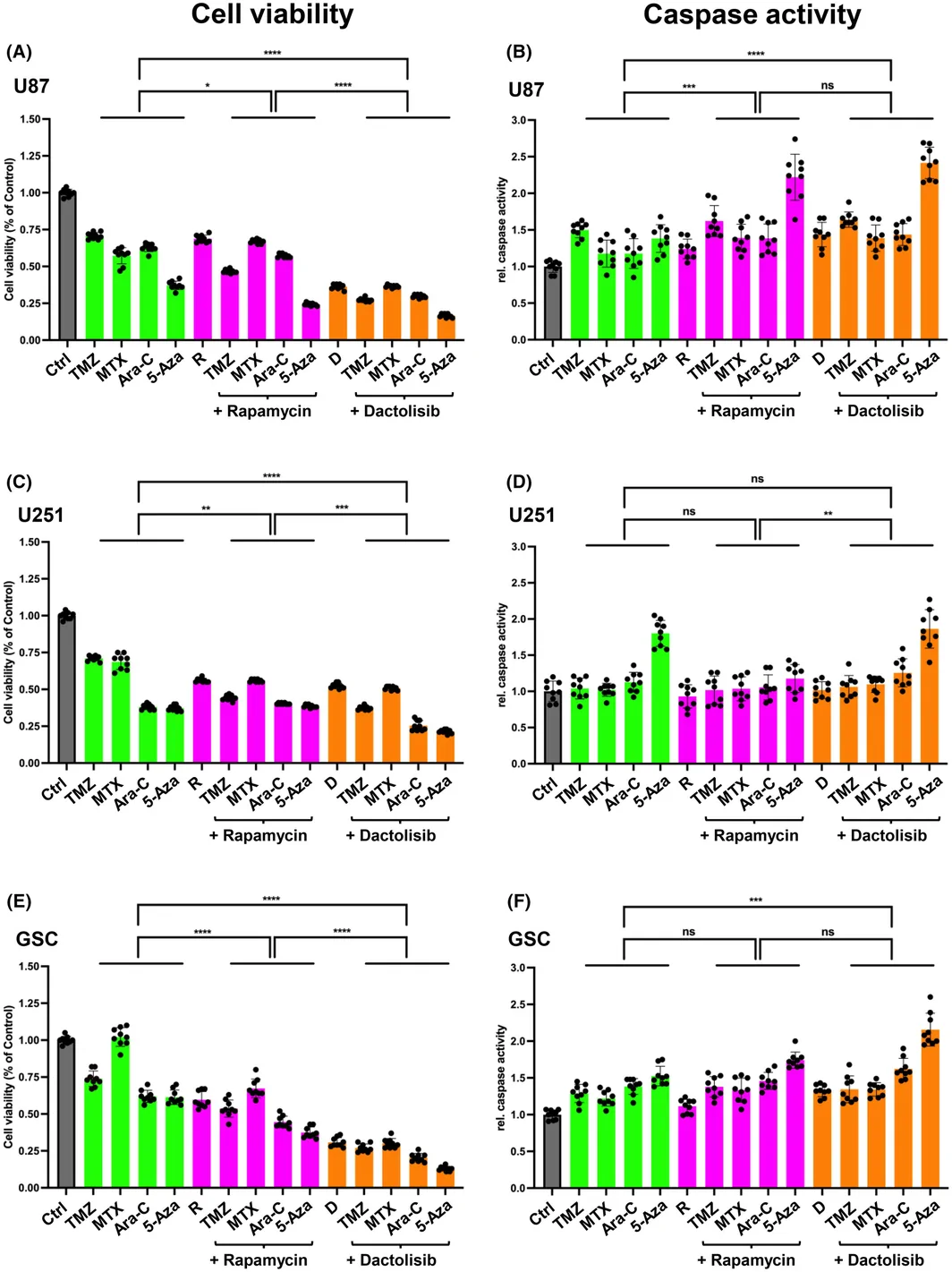
Cell viability and Caspase activity in U87, U251 and GSCs after combinatorial treatment with chemotherapeutic and either Rapamycin or Dactolisib. Each chemotherapeutic agent was combined pairwise with either Rapamycin or Dactolisib. U87 (TMZ 750 μm, MTX 50 nm, Ara‐C 8.5 μm, 5‐Aza 10 μm, Rapamycin 10 μm, Dactolisib 250 nm) and U251 (TMZ 100 μm, MTX 50 nm, Ara‐C 2.5 μm, 5‐Aza 10 μm, Rapamycin 10 μm, Dactolisib 250 nm) were treated for 72 h (viability) and 24 h (caspase). GSCs (TMZ 20 μm, MTX 50 nm, Ara‐C 500 nm, 5‐Aza 10 μm, Rapamycin 1 μm, Dactolisib 100 nm) were treated for 10 days (viability) and 48 h (caspase). All values are normalized to vehicle controls. Chemotherapeutics were pooled for statistical analysis. Data represent mean ± SD from three independent experiments in triplicates. One‐way ANOVA with *post hoc* (Tukey HSD) test was performed. *P < 0.05, **P < 0.01, ***P < 0.001, ****P < 0.0001, ns = not significant.

### Monotherapy with BH3‐mimetics (A1210477, Venetoclax), Eltanexor, or PAM pathway inhibitors (Rapamycin, Dactolisib) modulate the transcriptional expression of Bcl‐2, Mcl‐1, XPO1, mTOR, PIK3CA, and AKT2

These analyses were not intended as a target‐engagement assay: Rapamycin and Dactolisib act on catalytic activity and not on transcription. They map instead how the transcriptional program of the apoptotic machinery and of the PAM nodes is remodeled under treatment, thereby identifying the compensatory responses that motivate the combination. 5‐Aza was not included here and the following expression analyses because it acts on neither of these nodes. We analyzed the transcriptional effects of BH3‐mimetics (A1210477, Venetoclax), the XPO1‐inhibitor Eltanexor, and PAM pathway inhibitors (Rapamycin, Dactolisib) on mTOR, AKT2, PIK3CA, Bcl‐2, Mcl‐1, and XPO1 in U87, U251, and GSCs via qPCR (Fig. 3A,C, Fig. S2A). A1210477 downregulated mTOR in U87/U251 but not in GSCs; PIK3CA, Bcl‐2, and Mcl‐1 were strongly upregulated in GSCs. XPO1 was consistently downregulated across all models. Venetoclax induced strong Mcl‐1 upregulation in all cells, consistent with its known resistance mechanism [55, 56], reduced mTOR, AKT2, and PIK3CA in U87/U251, but increased PIK3CA in GSCs.

**Fig. 3:**
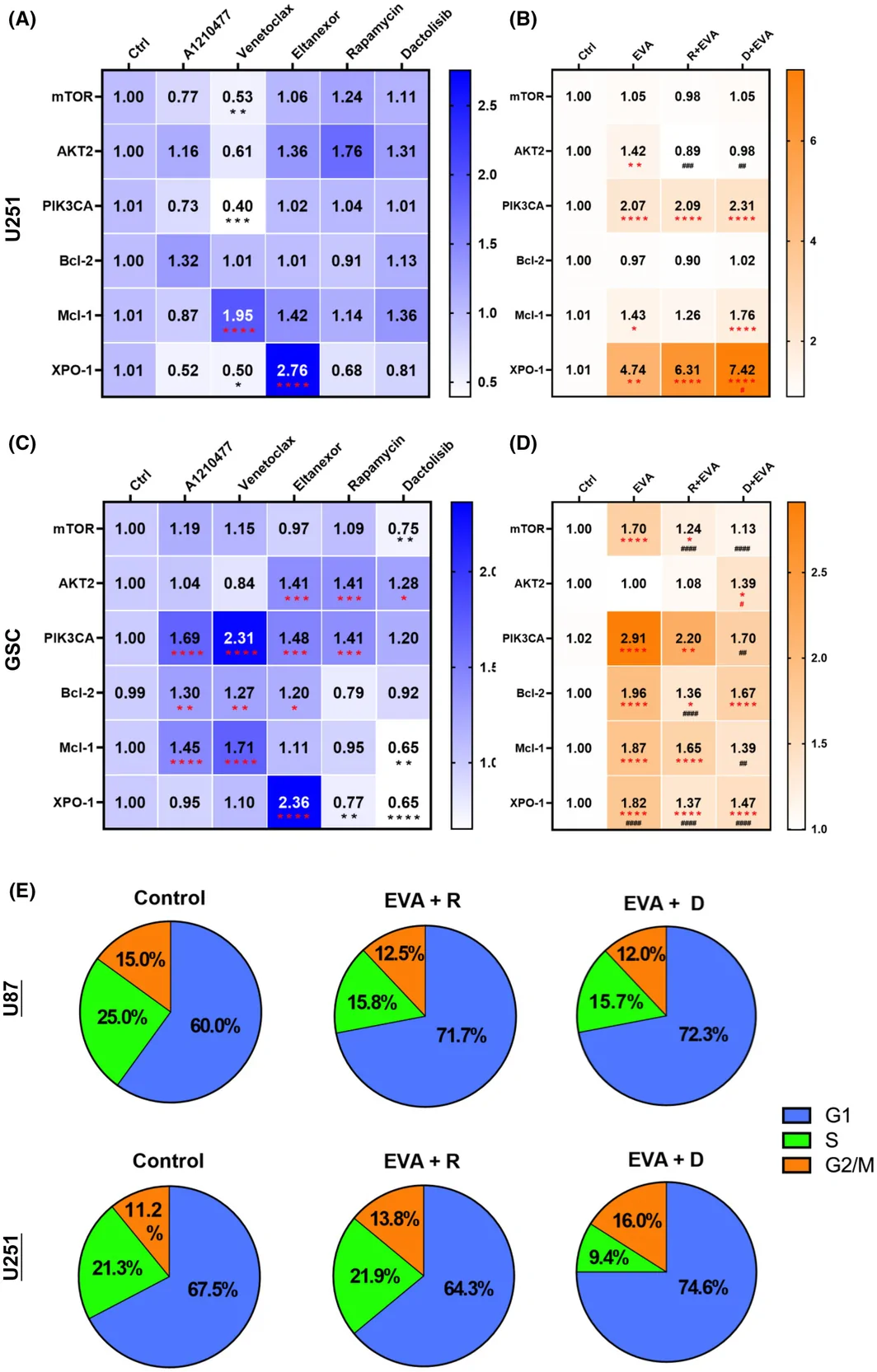
(A–D) Relative mRNA expression levels of mTOR, AKT2, PIK3CA, Bcl‐2, Mcl‐1, and XPO1 following treatment with A1210477, Venetoclax, Eltanexor, Rapamycin, Dactolisib, or EVA (Eltanexor, Venetoclax, A1210477) in combination with Rapamycin or Dactolisib. These panels report treatment‐induced changes of the transcriptional program of the apoptotic machinery and of the PAM nodes; they are not a target‐engagement assay. 5‐Aza is not included because it acts on neither of these nodes. U251 cells were treated for 24 h (A1210477: 1 μm; Venetoclax: 1 μm; Eltanexor: 100 nm; Rapamycin: 10 μm; Dactolisib: 250 nm), and GSCs for 48 h (A1210477: 100 nm; Venetoclax: 100 nm; Eltanexor: 10 nm; Rapamycin: 1 μm; Dactolisib: 100 nm). Red indicates upregulation, black indicates downregulation. Expression levels are based on mean values from three independent experiments relative to untreated controls. * = vs. control, # = vs. EVA. */#*P* < 0.05, **/##*P* < 0.01, ***/###*P* < 0.001; ****/####*P* < 0.0001; ns = not significant. (E) Cell cycle distribution was analyzed by flow cytometry following PI staining. U87 and U251 cells were treated for 24 h (A1210477: 1 μm; Venetoclax: 1 μm; Eltanexor: 100 nm; Rapamycin: 10 μm; Dactolisib: 250 nm). Pie charts illustrate the proportions of cells in G1 (blue), S (green), and G2/M (orange) phases. Representative experiments of three independent experiments are shown; as a pie chart cannot display dispersion, the mean ± SD of all three experiments are given in the section “Treatment with EVA and PAM pathway inhibitors induces cell cycle arrest in glioblastoma cells” below.

Eltanexor increased XPO1, Bcl‐2, and Mcl‐1 in U251 and GSCs, confirming previous findings [37], but downregulated mTOR, AKT2, and PIK3CA in p53‐wild‐type U87, while upregulating AKT2 and PIK3CA in p53‐mutant U251 and GSCs, suggesting p53‐dependent regulation [57]. Dactolisib more effectively reduced mTOR, AKT2, and PIK3CA in U87 and mTOR in GSCs. However, both inhibitors increased AKT2 and PIK3CA in U251 and GSCs, indicating transcriptional compensation rather than pathway activation. Bcl‐2 remained largely unchanged, but Mcl‐1 and XPO1 were significantly reduced in U87 and GSCs, suggesting a shift towards a pro‐apoptotic transcriptional profile.

### Combinatorial treatment with EVA and Rapamycin or Dactolisib modulates Bcl‐2, Mcl‐1, XPO1, mTOR, PIK3CA, and AKT2 expression at transcriptional and protein level

To evaluate molecular responses to combined EVA treatment with or without Rapamycin or Dactolisib, we analyzed Bcl‐2, Mcl‐1, XPO1, mTOR, PIK3CA, and AKT2 mRNA expression via qPCR (Fig. 3B,D, Fig. S2B) and western blot (Figs S3 and S4) in U87, U251, and GSCs. EVA upregulated Mcl‐1 and XPO1 mRNA in all models, with strongest effects upon addition of Venetoclax compared to E or A alone (Fig. 3B,D, Fig. S2B), paralleling Mcl‐1 protein increase, particularly in U251, likely due to A1210477‐mediated protein stabilization [58]. In GSCs, mRNA upregulation did not translate to protein changes, indicating post‐transcriptional regulation. EVA increased mTOR, AKT2, and PIK3CA mRNA in the majority, but not in all, of the model–target combinations examined (Fig. 3B,D, Fig. S2B); the induction of PAM transcripts was therefore consistent in direction but not uniform. These transcript changes were not mirrored at the protein level (Fig. S4), suggesting post‐translational repression, notably for PIK3CA. Co‐treatment with Rapamycin or Dactolisib further enhanced Mcl‐1 and XPO1 mRNA especially in GSCs (Fig. 3D), while XPO1 protein decreased with EVA + Dactolisib in U87/U251 (Fig. S3A–D), indicating transcriptional compensation. Bcl‐2 mRNA was stable or increased, but protein consistently downregulated, supporting a shift towards apoptosis. In U251, Mcl‐1 increased at both levels under EVA and EVA + Dactolisib, again suggesting A1210477‐induced stabilization [58]. PIK3CA mRNA levels rose under EVA + Rapamycin or Dactolisib (Fig. 3B,D, Fig. S2B), but protein declined in U87/U251, especially under Dactolisib (Fig. S4A–D). AKT2 mRNA dropped (U87/U251; Fig. S2B, Fig. 3B), while protein increased slightly (Fig. S4A–F). mTOR protein decreased in U87 and GSCs under EVA + Dactolisib (Fig. S4A,B and E,F), matching mRNA decrease in U87, but not in GSCs. In U251, mTOR remained unchanged at both levels. The two PAM inhibitors differed markedly at the protein level (Fig. S4): none of the three PAM components changed significantly in the Rapamycin arm in any of the three cell models, whereas under Dactolisib, PIK3CA was reduced in U87 (*P* < 0.0001) and U251 (*P* < 0.05) and mTOR in U87 (*P* < 0.05) and in GSCs (*P* < 0.01); AKT2 was not reduced in any model. The same asymmetry was apparent at the transcript level in the patient‐derived GSCs, in which Dactolisib attenuated the EVA‐induced increase in mTOR and PIK3CA significantly more than Rapamycin (Fig. 3D).

### Treatment with EVA and PAM pathway inhibitors induces cell cycle arrest in glioblastoma cells

To assess cell cycle effects of BH3‐mimetics (A1210477, Venetoclax), Eltanexor, and PAM pathway inhibitors (Rapamycin, Dactolisib), U87 (Fig. 3E, Fig. S5A) and U251 (Fig. 3E, Fig. S5B) cells were treated for 24 h with either EVA, Rapamycin, Dactolisib, or their combinations. PI staining revealed a shift towards G1 phase under Dactolisib monotherapy in both cell lines, accompanied by a lower S‐phase fraction, whereas the combinations with EVA did not raise the G1 fraction above control level. Rapamycin produced a comparable G1 shift in U251, but not consistently in U87. The reduction of the S‐phase fraction under Dactolisib indicates impaired S‐phase progression and a potential linkage to apoptosis induction [59]. Across the three independent experiments the cell cycle distributions were, as mean ± SD for each treatment group and phase: in U87 cells, the G1/S/G2–M distribution was 60.2 ± 0.5/23.5 ± 1.8/16.3 ± 1.3% in untreated controls, 56.4 ± 10.5/33.1 ± 14.3/10.5 ± 3.9% under EVA, 57.4 ± 15.2/26.7 ± 17.2/15.9 ± 5.8% under Rapamycin, 62.1 ± 10.2/25.7 ± 10.7/12.2 ± 0.6% under EVA + Rapamycin, 67.1 ± 3.8/18.5 ± 7.6/14.3 ± 5.9% under Dactolisib and 59.2 ± 12.8/29.7 ± 13.1/11.0 ± 1.4% under EVA + Dactolisib. In U251 cells, the corresponding values were 71.1 ± 3.1/17.5 ± 3.8/11.4 ± 1.8% (control), 64.7 ± 1.9/17.5 ± 3.3/17.8 ± 4.8% (EVA), 78.0 ± 1.8/12.7 ± 0.8/9.3 ± 1.2% (Rapamycin), 64.7 ± 1.8/20.6 ± 1.3/14.7 ± 1.3% (EVA + Rapamycin), 78.4 ± 2.6/13.1 ± 1.8/8.5 ± 1.1% (Dactolisib) and 70.1 ± 4.1/15.3 ± 5.6/14.6 ± 1.6% (EVA + Dactolisib). The larger standard deviations in U87 reflect inter‐experimental variability in the partition between G1 and S phase. Since a pie chart cannot display dispersion, Fig. 3E and Fig. S5 show representative experiments and the averaged values are given here.

### Evaluation of cell viability and caspase activity upon combined treatment with chemotherapeutics (TMZ, MTX, Ara‐C, 5‐Aza), EVA, and either Rapamycin or Dactolisib in glioblastoma cell lines and GSCs

Given the limitations of monotherapy in glioblastoma, we explored combinatorial strategies involving apoptosis‐inducing chemotherapeutics (TMZ, MTX, Ara‐C, or 5‐Aza), BH3‐mimetics (V, A), the XPO1 inhibitor Eltanexor, and PAM pathway inhibitors (Rapamycin or Dactolisib). Cell viability was first assessed via DAPI staining (Fig. S6), followed by CellTiter‐Glo and Caspase‐Glo 3/7 assays with sublethal drug doses. EVA alone slightly increased caspase activity without affecting viability, indicating apoptotic priming. Combining chemotherapeutics with EVA significantly enhanced cytotoxicity and apoptosis across models, except MTX in GSCs (Fig. 4A,C,E), with viability loss consistently linked to caspase activation (Fig. 4B,D,F). EVA co‐treatment with Rapamycin or Dactolisib further reduced viability and increased apoptosis in U87 and GSCs, but apoptosis was not affected in U251. EVA + Dactolisib combinations were consistently more effective than EVA + Rapamycin (Fig. 4A–F). Notably, 5‐Aza combined with EVA, EVA + Rapamycin, or EVA + Dactolisib induced apoptosis across all models, without significant differences; however, EVA + Dactolisib + 5‐Aza showed the strongest cytotoxicity, suggesting involvement of non‐apoptotic cell death mechanisms. To address the relationship between apoptosis induction and loss of viability formally, relative caspase‐3/7 activity was correlated with relative cell viability across all treatment groups of each cell line (Spearman rank correlation): U87, *r* = −0.882, *P* < 0.0001; U251, *r* = −0.569, *P* = 0.017; GSCs, *r* = −0.583, *P* = 0.014 (*n* = 17 treatment groups per cell line). The corresponding caspase‐to‐viability ratios of the individual combination treatments rose in parallel with treatment efficacy in U87 (Rapamycin 2.0, EVA + Rapamycin 5.2, Dactolisib 6.6, EVA + Dactolisib 52.1, TMZ + EVA + Dactolisib 76.5, 5‐Aza + EVA + Dactolisib 469.4) and in GSCs (1.8, 2.9, 4.8, 7.4, 6.5 and 30.8 for the same treatment arms), whereas in U251 they remained within a narrow range (1.6–3.1 for the PAM pathway inhibitor arms, 21.0 for 5‐Aza + EVA + Dactolisib).

**Fig. 4:**
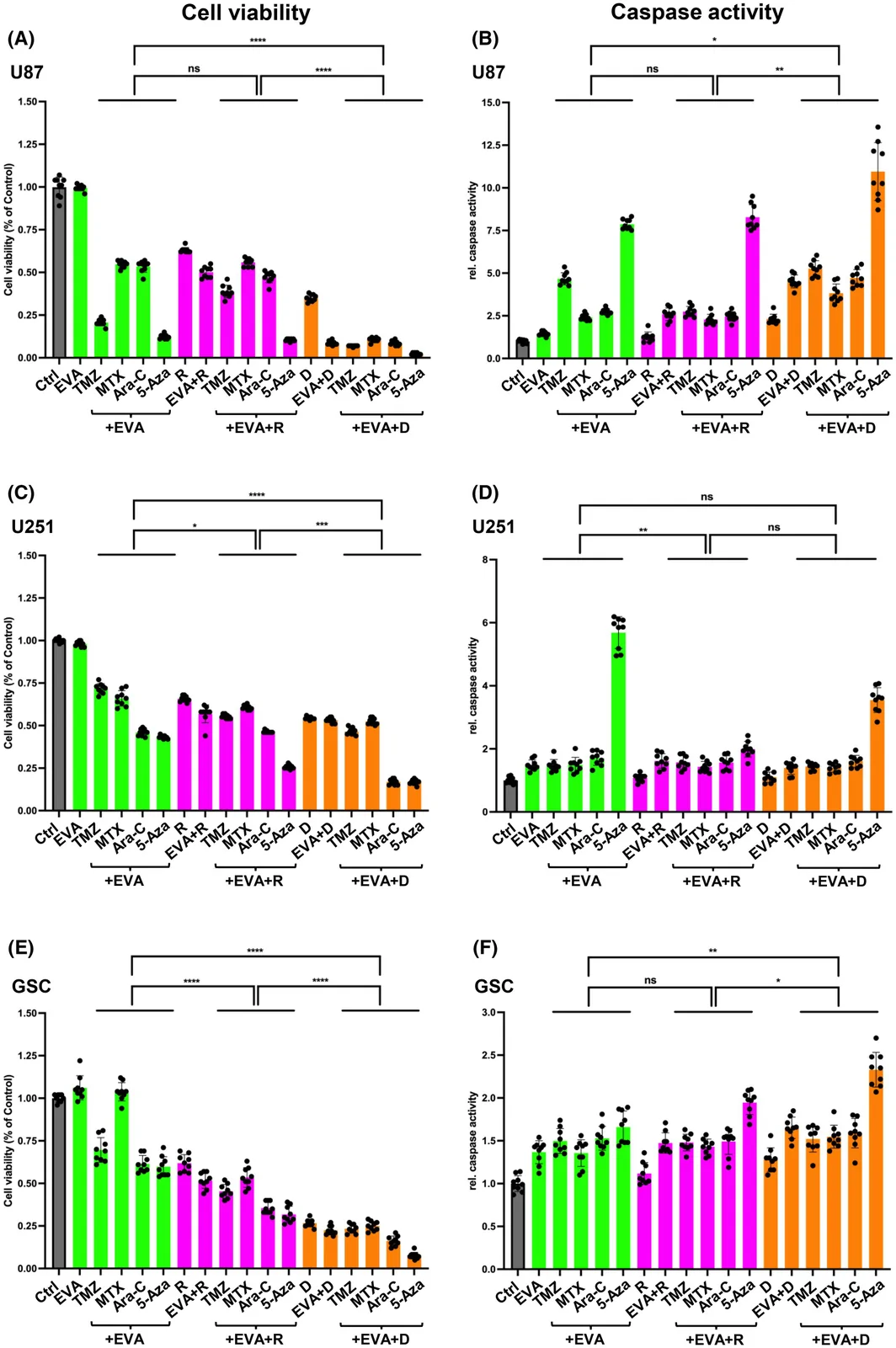
Cell viability (A, C, E) and Caspase activity (B, D, F) following combinatorial treatment with chemotherapeutic agents, EVA, and either Rapamycin or Dactolisib in U87 cells (A, B), U251 cells (C, D), and GSCs (E, F). Panels A–F show relative cell viability (A, C, E) and caspase activity (B, D, F) in U87, U251, and GSCs following treatment with chemotherapeutics (TMZ, MTX, Ara‐C, 5‐Aza), EVA (Eltanexor, Venetoclax, A1210477), and either Rapamycin or Dactolisib. U87 (TMZ 750 μm, MTX 50 nm, Ara‐C 8.5 μm, 5‐Aza 10 μm, R 10 μm, D 250 nm, A/V 1 μm, E 100 nm), U251 (TMZ 100 μm, MTX 30 nm, Ara‐C 2.5 μm, others as above), GSCs (TMZ 20 μm, MTX 50 nm, Ara‐C 500 nm, 5‐Aza 10 μm, R 1 μm, D 100 nm, A/V 100 nm, E 10 nm). Chemotherapeutics were pooled for statistical analysis. Data represent mean ± SD from three independent experiments in triplicates. One‐way ANOVA with a consecutive *post hoc* test (Tukey HSD) was used to analyze, *P < 0.05, **P < 0.01, ***P < 0.001, ****P < 0.0001, ns: not significant. Relative caspase‐3/7 activity and relative cell viability were inversely correlated across the 17 treatment groups of each cell line (Spearman rank correlation: U87 *r* = −0.882, *P* < 0.0001; U251 *r* = −0.569, *P* = 0.017; GSCs *r* = −0.583, *P* = 0.014).

### Autophagy‐related protein modulation following combined treatment with chemotherapeutics, EVA, and Rapamycin or Dactolisib in glioblastoma cell lines and GSCs

We analyzed LC3I‐to‐LC3II conversion and p62 levels by western blot as markers of autophagosome formation and autophagic flux in U87, U251, and GSCs (Fig. S7A–F). In U87 and U251, all combination treatments increased LC3II (Fig. S7A–D), whereas in GSCs, conversion occurred only with chemotherapeutics combined with EVA plus Rapamycin or Dactolisib (Fig. S7E–F). p62 was elevated across all treatments, indicating impaired autophagic flux. Notably, 5‐Aza + EVA + Dactolisib led to the strongest LC3II accumulation alongside persistently elevated p62 in all models, indicating pronounced autophagy blockade. Despite highest cytotoxicity (Fig. 4A,C,E), caspase activity in this group was similar to 5‐Aza + EVA or 5‐Aza + EVA + Rapamycin (Fig. 4B,D,F), suggesting that impaired autophagic flux may contribute to cell death in concert with apoptosis.

### Immunofluorescence staining of apoptotic and dead cells in mouse brain slices treated with 5‐Aza, Rapamycin, Dactolisib, and EVA alone or in combination

Cultured mouse brain slices were treated with 5‐Aza, Rapamycin, Dactolisib, and EVA alone or in combination. Apoptosis and cell death were assessed by Caspase‐3 and PI immunofluorescence, with Hoechst for nuclear staining and Staurosporine as positive control. Only Staurosporine‐treated slices showed strong Caspase‐3 and PI signals; the treatment groups showed no substantial signal enhancement compared to untreated controls (Fig. 5).

**Fig. 5:**
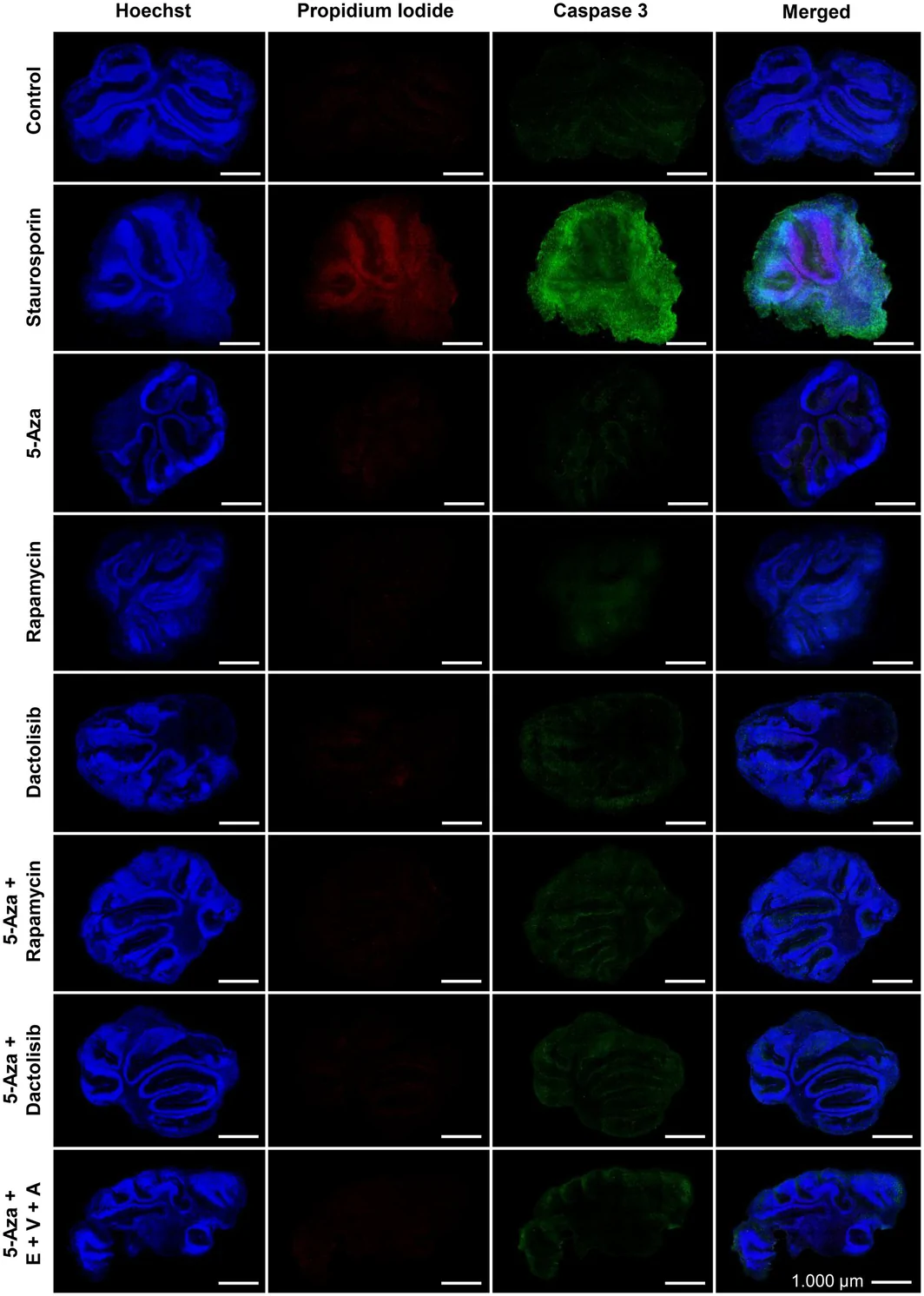
Neurotoxicity assessment by immunofluorescence staining in organotypic mouse brain slice cultures. Slices were treated with 5‐Aza, Rapamycin, or Dactolisib as monotherapies, or with 5‐Aza in combination with EVA (Eltanexor + Venetoclax + A1210477), Rapamycin, or Dactolisib. Staurosporine and DMSO served as positive and negative controls, respectively. Dead cells were visualized by propidium iodide (PI, red), apoptotic cells by Caspase‐3 staining (green), and nuclei by Hoechst staining (blue). Scale bars indicate 1000 μm.

### EVA combined with PAM‐inhibitors suppress tumor growth and KI67‐expression in an *ex vivo* CAM model

To evaluate the effects of EVA combined with Rapamycin or Dactolisib on tumor growth, angiogenesis and embryo development, we used a chicken embryo xenograft model [52]. After tumor establishment, treatment was applied for 3 days (Fig. 6A). [^18^F]FDG‐PET/CT imaging demonstrated suppression of tumor metabolic activity in EVA‐based combination regimens (Fig. 6B,D). Ki‐67 immunohistochemistry served as the primary histological readout, with the adjacent H&E sections providing the morphological context (Fig. 6C). Ki‐67 was clearly decreased in combination‐treated xenografts. Under Rapamycin, regressive and partly necrotic areas coexisted with preserved, Ki‐67‐positive vital tumor nests within the same xenograft, whereas such nests were almost entirely absent under Dactolisib; morphological regression alone therefore does not indicate loss of proliferative capacity. Western blot confirmed Caspase‐3 activation and PARP cleavage exclusively with EVA + Dactolisib (Fig. 6E–H), indicating synergistic antitumor and pro‐apoptotic effects in U87 CAM tumors, even at sublethal doses.

**Fig. 6:**
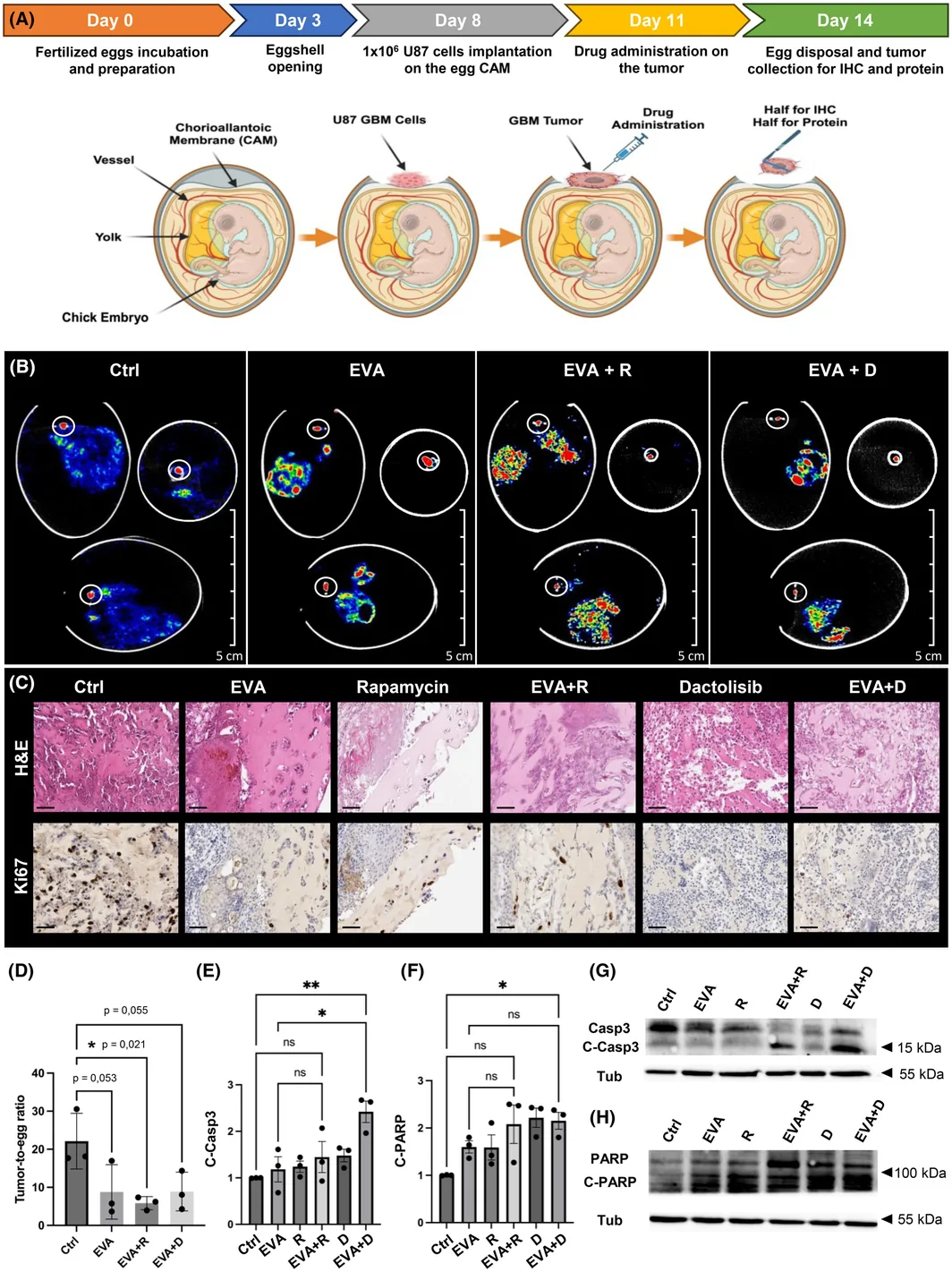
EVA combined with PAM inhibitors (Rapamycin or Dactolisib) suppresses tumor metabolic activity and modulates apoptosis in a chicken chorioallantoic membrane xenograft model. (A) Schematic representation of the experimental procedure. (B) Representative [^18^F]FDG PET/CT images shown in coronal, axial, and sagittal planes for each treatment group (Untreated, Rapamycin, Dactolisib, EVA, EVA + Rapamycin, EVA + Dactolisib). The color bar represents [^18^F]FDG distribution as %ID·mL^−1^; red indicates high uptake, blue indicates low uptake. The white silicone ring delineates the xenograft area. Scale bars indicate 5 cm. (C) Ki‐67 immunohistochemistry is the primary histological readout; the adjacent H&E sections provide the morphological context. Under Rapamycin, Ki‐67‐positive vital tumor nests persist within regressive, partly necrotic areas, whereas such nests are almost entirely absent under Dactolisib. Scale bars indicate 50 μm. (D) Tumor‐to‐whole‐egg [^18^F]FDG uptake ratio for each treatment group. Data represent mean ± SD; *n* = 3 eggs per group; *P < 0.05. (E) Quantification of cleaved Caspase‐3 relative to Tubulin by western blot. Data represent mean ± SEM from three independent experiments; *P < 0.05, ns: not significant. (F) Quantification of cleaved PARP relative to Tubulin by western blot. Data represent mean ± SEM from three independent experiments; *P < 0.05, ns: not significant. One‐way ANOVA with *post hoc* (Tukey HSD) test was performed for (D–F). (G) Representative western blot strips for cleaved Caspase‐3 and Tubulin. (H) Representative western blot strips for cleaved PARP and Tubulin. The complete, uncropped membranes including molecular weight markers are provided in the supplementary material, in which the region shown in each panel is outlined.

### [ ^18^F]FDG‐PET/CT imaging of U87 xenografts in the chick chorioallantoic membrane model

To assess the metabolic impact of treatment on tumor xenografts, [^18^F]FDG‐PET/CT imaging was performed following 3 days of drug exposure (Fig. 6B). The tumor‐to‐whole‐egg [^18^F]FDG uptake ratio served as the primary quantitative endpoint to account for inter‐individual variability in radiotracer injection efficiency (coefficient of variation approximately 47%; Fig. 6D; Table 2). One‐way ANOVA revealed a significant overall treatment effect (*F*(3, 8) = 4.77, *P* = 0.034). Untreated controls showed a mean tumor‐to‐egg ratio of 22.13 ± 7.31. EVA + Rapamycin reduced this to 5.84 ± 1.74 (*P* = 0.021 vs. controls, Tukey HSD *post hoc* analysis), EVA monotherapy to 8.79 ± 7.15 (*P* = 0.055), and EVA + Dactolisib to 8.90 ± 5.08 (*P* = 0.053).

**Table 2:** [^18^F]FDG‐PET/CT quantification of U87 xenografts in the chick chorioallantoic membrane (CAM) model. Individual values for *n* = 3 biological replicates per group. The tumor‐to‐egg ratio normalizes tumor [^18^F]FDG uptake (%ID/mL) to whole‐egg uptake to correct for inter‐individual variability in injection efficiency. EVA = Eltanexor + Venetoclax + A1210477; EVA + R = EVA + Rapamycin; EVA + D = EVA + Dactolisib.

Group	Tumor (%ID·mL^−1^)	Egg (%ID·mL^−1^)	Tumor‐to‐egg ratio (plotted)
Ctrl	324 644	17 917	181 193
Ctrl	713 232	23 338	305 610
Ctrl	375 945	21 241	176 990
EVA	462 174	27 264	169 518
EVA	102 093	27 921	36 565
EVA	114 792	19 922	57 621
EVA + R	120 733	19 418	62 176
EVA + R	210 059	28 534	73 617
EVA + R	92 087	23 310	39 505
EVA + D	431 030	30 032	143 524
EVA + D	137 171	17 006	80 660
EVA + D	122 625	28 590	42 891

## Discussion

### Combination of chemotherapeutics with EVA and PAM‐inhibition

Previous work from our laboratory demonstrated the efficacy of combining sublethal doses of XPO1, Bcl‐2 and Mcl‐1 inhibitors (Eltanexor, Venetoclax, and A1210477; EVA) with individual chemotherapeutics (TMZ, MTX, or Ara‐C) to overcome glioblastoma survival mechanisms and restore apoptotic signaling in order to fully execute the apoptotic potential in treated tumor cells [36, 37]. Observations of compensatory responses, such as Mcl‐1 upregulation following Bcl‐2 inhibition [55] and marked induction of XPO1 upon EVA treatment, led to the hypothesis that additional resistance pathways, notably the PAM axis, may contribute to therapeutic escape from the EVA combination therapy. In glioblastoma, hyperactivation of PI3K/Akt signaling commonly results from PIK3CA amplification or gain‐of‐function mutations, as well as PTEN loss‐of‐function alterations, e.g. 10q loss, a frequent genetic alteration in glioblastoma [60]. Initial experimental and literature‐based analyses revealed significant Akt upregulation in glioblastoma tissue compared to normal brain, whereas PIK3CA showed a trend towards higher expression only. Immunohistochemical data further confirmed preferential expression of these proteins in glioblastoma tumor cells. All glioblastoma cell lines tested, particularly GSCs, exhibited marked overexpression of PAM pathway genes. Moreover, EVA treatment induced transcriptional upregulation of PAM components in most, although not in all, models and targets, supporting the hypothesis that PAM signaling serves as a key adaptive resistance mechanism to EVA therapy. The interpretation of this study does not, however, rest on the tissue‐level comparison in Fig. 1A–C, which serves to place our models in the context of published cohort data; the functional argument rests on the expression data obtained in the cell models used here (Fig. 1H–J) and on the treatment experiments that follow. The discrepancy between mRNA and protein expression highlights the role of post‐transcriptional regulation in drug‐treated glioblastoma cells. Large‐scale proteogenomic work shows that mRNA levels only partially predict the amount of protein because translation rates and protein half‐lives vary substantially across genes and conditions [61, 62]. Pharmacological PI3K/mTOR blockade (Rapamycin/Dactolisib) suppresses cap‐dependent translation via the S6K1/4EBP axis and stress signaling, so EVA‐induced transcriptional upregulation of PAM components does not necessarily result in higher protein levels [24]. Conversely, Mcl‐1–directed BH3 mimetics can stabilize Mcl‐1 protein by reducing its ubiquitination and enhancing de‐ubiquitination, explaining elevated Mcl‐1 protein despite variable mRNA [58]. Notably, such transcript–protein decoupling is well documented in glioblastoma proteogenomic cohorts [63] and, together with our findings (Fig. 3A–D, Figs S2–S4), supports the interpretation that EVA + Dactolisib achieves functional suppression of the PAM axis at the protein level despite compensatory transcriptional feedback, underlining the need to evaluate both mRNA and protein endpoints.

In this study, we used chemotherapeutic agents (TMZ, MTX, Ara‐C, or 5‐Aza) as inducers of apoptosis, in combination with the EVA regimen and PAM pathway blockade, to overcome glioblastoma cell survival mechanisms. We found that Dactolisib (a dual PI3K/mTOR inhibitor) was significantly more effective than the mTORC1 inhibitor Rapamycin. Dactolisib plus EVA reduced cell viability and induced robust apoptosis in glioblastoma cell lines and GSCs, whereas Rapamycin plus EVA was less potent and even upregulated certain survival genes in GSCs. Notably, Dactolisib caused G1 cell cycle arrest and synergized with EVA to trigger caspase‐3 activation and PARP inactivation in an *ex vivo* chicken embryo model, without evident toxicity to normal mouse brain tissue. These findings underscore that comprehensive PAM pathway inhibition, targeting both PI3K and mTOR, markedly enhances the pro‐apoptotic efficacy of EVA plus chemotherapeutics, highlighting a potent combination strategy for glioblastoma therapy.

These findings were further corroborated by [^18^F]FDG‐PET/CT imaging of U87 CAM xenografts, with metabolic activity quantified as the tumor‐to‐egg ratio to normalize for inter‐egg variability in tracer distribution (Table 2). One‐way ANOVA revealed a significant overall treatment effect across all groups (*F*(3, 8) = 4.77, *P* = 0.034), with all treated groups showing markedly reduced [^18^F]FDG uptake relative to untreated controls (mean tumor‐to‐egg ratio: 22.13 ± 7.31). EVA + Rapamycin achieved the strongest metabolic suppression, with a mean ratio of 5.84 ± 1.74; a reduction of more than 70% compared to controls, reaching statistical significance (*P* = 0.021). EVA monotherapy and EVA + Dactolisib demonstrated similar substantial reductions (mean ratios 8.79 ± 7.15 and 8.90 ± 5.08, respectively), with both approaching significance (*P* = 0.055 and *P* = 0.053). Collectively, these imaging data strongly support the conclusion that combining EVA with PAM pathway inhibition effectively suppresses tumor metabolic activity *in vivo*, reflecting impaired glycolysis as a consequence of multi‐target apoptosis induction.

### Rapamycin vs Dactolisib: PAM Pathway inhibition and feedback loops

The superior performance of Dactolisib over Rapamycin can be attributed to a more complete blockade of the PAM pathway and mitigation of compensatory feedback loops. While Rapamycin inhibits mTORC1, it does not block PI3K or mTORC2, and partial PAM inhibition often triggers compensatory activation of upstream survival signals such as activation of Akt [64]. Indeed, clinical studies in PTEN‐deficient glioblastoma reported that Rapamycin treatment led to an increased Akt activation, presumably due to loss of negative feedback [65]. Consistent with this, Rapamycin is significantly less potent in our glioblastoma models and instead elicited upregulation of pro‐survival genes, reflecting an adaptive resistance. In contrast, Dactolisib concurrently targets PI3K and mTOR, preventing Akt re‐activation and more completely silencing PAM signaling. This is in line with prior studies showing dual PI3K/mTOR inhibitors induce G1 arrest and apoptosis in glioblastoma cells more effectively [66]. Notably, Dactolisib did not provoke the same degree of Akt‐driven feedback; instead, it significantly downregulated mTOR transcription in U87 and GSCs.

This mechanistic distinction was further reflected in the metabolic imaging data: both EVA + Rapamycin and EVA + Dactolisib markedly reduced tumor‐to‐egg ratios compared to untreated controls (5.84 ± 1.74 and 8.90 ± 5.08, respectively), with no statistically significant difference between the two combination regimens (*P* = 0.913). While EVA + Rapamycin showed a numerically lower mean ratio, the high within‐group variance in the EVA + Dactolisib group, likely attributable to the limited sample size of this pilot cohort, precludes definitive conclusions regarding differential efficacy from the imaging data alone. The superior performance of Dactolisib over Rapamycin observed in the *in vitro* and *ex vivo* models is therefore interpreted in the broader context of the mechanistic and viability data presented above:

While EVA + Rapamycin achieved the most statistically robust metabolic suppression in the PET endpoint (*P* = 0.0216), apoptotic markers measured by western blot showed significant induction of cleaved Caspase‐3 and cleaved PARP exclusively with EVA + Dactolisib (Fig. 6E–H). This divergence indicates that EVA + Rapamycin acts predominantly through cytostatic mechanisms, reducing metabolic activity without triggering frank apoptosis, whereas EVA + Dactolisib combines metabolic suppression with cytotoxic, pro‐apoptotic signaling. Such a distinction between cytostatic and cytotoxic drug action has direct implications for clinical treatment planning: cytostatic regimens may achieve disease stabilization, whereas cytotoxic regimens offer the potential for tumor regression. The CAM‐PET model thus enables functional discrimination between these mechanistic profiles in a preclinical setting. The histological findings point in the same direction: under Rapamycin, Ki‐67‐positive vital tumor nests persisted within regressive, partly necrotic areas of the same xenograft, whereas such nests were almost entirely absent under Dactolisib (Fig. 6C). This spatially heterogeneous, predominantly cytostatic effect of Rapamycin contrasts with the cytotoxic effect of Dactolisib, and it shows that morphological regression alone does not indicate loss of proliferative capacity.

Furthermore, glioblastoma therapies must balance effective tumor targeting with preservation of immune competence, a prerequisite for successful long‐term control. Rapamycin (Sirolimus) exerts anti‐proliferative effects but is also strongly immunosuppressive. It suppresses T‐cell proliferation and cytokine signaling, expanding regulatory T cells and impairing antitumor immunity. This is especially problematic in glioblastoma, where immune evasion is a significant hallmark. Clinically, Rapamycin is primarily used as an immunosuppressant to prevent organ rejection in patients undergoing organ transplantation and is linked to infection risk, impaired wound healing, and metabolic dysregulation, limiting its utility in immune‐sensitive oncology settings [67, 68].

Dactolisib yields superior antitumor effects in preclinical glioblastoma models [69, 70]. In contrast to Rapamycin, immunosuppressive effects of Dactolisib are significantly attenuated. It does not impair T‐cell responses to the same extent. Instead, it may enhance antitumor immunity and impair the viability and immunosuppressive function of myeloid‐derived suppressor cells (MDSCs) [71, 72]. Side effects of Dactolisib are driven by systemic toxicity (e.g., hepatotoxicity, GI toxicity) due to its pan‐PI3K inhibition [35]. Despite Dactolisib's higher toxicity at effective doses, its relative preservation of immune function makes it more compatible with emerging immunotherapeutic strategies in glioblastoma treatment, such as checkpoint inhibitors [71]. Especially when administered under circumvention of the blood–brain barrier, i.e. directly to the brain, systemic side effects of Dactolisib should be manageable.

### The dual role of autophagy and apoptosis in glioblastoma cell fate regulation

In all glioblastoma cells investigated, cell viability and caspase‐3/7 activity were inversely correlated across the treatment arms, most clearly in U87 (*r* = −0.882) and less strongly, although still significantly, in GSCs (*r* = −0.583) and U251 (*r* = −0.569). Apoptosis is therefore a relevant mechanism to underpin the therapeutic feasibility of the drug combinations used here, while the weaker coefficient in U251 corresponds to the limited caspase induction observed in this cell line. 5‐Aza in combination with either EVA or EVA plus Rapamycin or Dactolisib induced apoptosis across all models; however, no significant differences were observed between these treatment groups. Nonetheless, 5‐Aza in combination with EVA and Dactolisib produced the strongest cytotoxic response, suggesting the involvement of additional, non‐apoptotic cell death pathways. We observed that treatment with EVA and PAM inhibitors in combination with 5‐Aza induced autophagy‐related changes alongside apoptosis. In some contexts, impaired autophagic flux may contribute to cell death in concert with apoptosis. We noted concurrent LC3‐II accumulation and p62 elevation in cells treated with EVA plus Dactolisib and 5‐Aza, consistent with autophagy blockade, which may facilitate cell death beyond apoptosis alone. The potency of 5‐Aza is clearly distinct from that of TMZ, which induces a cell death mechanism in which apoptosis plays only a minor role, while senescence is the predominant outcome [73]. Considering the multitude of resistance mechanisms, particularly the role of senescence as a strategy to evade apoptosis in response to TMZ, it appears more promising to use combinatorial treatments that promote apoptosis and/or autophagy dysregulation rather than senescence.

However, autophagy is a double‐edged sword in cancer therapy. Tumor cells can activate autophagy as a survival mechanism under therapeutic stress, thereby recycling nutrients and averting apoptosis. This appears to occur with XPO1 inhibition: recent evidence indicates that Selinexor predominantly induces autophagy (rather than apoptosis) in glioblastoma cells, and that this autophagy provides a protective effect, since blocking autophagy significantly increases Selinexor‐induced cell death [74]. A similar phenomenon has been noted with mTORC1 inhibition, where autophagy upregulation and Akt feedback activation together allow cancer cells to resist apoptosis [75]. In our experiments, the comprehensive blockade of survival pathways by Dactolisib + EVA and 5‐Aza may impair autophagic flux, thereby preventing cells from utilizing autophagy as a cytoprotective mechanism, or alternatively, apoptosis induction is so dominant that any pro‐survival autophagy is insufficient to rescue the cells. Indeed, the net outcome was massive cell death in Dactolisib + EVA + 5‐Aza‐treated tumors.

### Targeting GSCs: A key strategy in overcoming glioblastoma resistance

GSCs represent the primary tumor subpopulation responsible for chemoresistance and exhibit a strong capacity for self‐renewal, both of which contribute significantly to glioblastoma recurrence. GSCs in our models had especially high baseline PIK3CA and AKT expression, and they were less responsive to Rapamycin, consistent with the view that GSCs activate potent survival programs. These stem‐like cells can rapidly adapt to single‐pathway inhibition and often resist apoptosis, contributing to tumor relapse [76]. Conventional therapies frequently fail to eliminate GSCs, which then, based on the tumor stem cell hypothesis, regenerate the tumor [77]. Encouragingly, the combination of Dactolisib with EVA and 5‐Aza most effectively induced apoptosis and reduced cell viability in GSC cultures at doses that are sublethal when given individually. These findings suggest that simultaneously disrupting multiple survival pathways, mTOR/PI3K signaling, nuclear export, and Bcl‐2/Mcl‐1‐mediated apoptosis resistance, can overcome the intrinsic therapy resistance of GSCs. Moreover, the absence of significant toxicity in mouse brain slice cultures and the CAM model indicates that higher doses of EVA and Dactolisib might be feasible.

Importantly, [^18^F]FDG‐PET/CT imaging of treated CAM xenografts provided functional confirmation that the observed viability reduction translates into measurable metabolic suppression at the whole‐tumor level, bridging the gap between *in vitro* apoptosis assays and a preclinical imaging readout with direct clinical relevance, as [^18^F]FDG‐PET is routinely used to monitor treatment response in neuro‐oncology. The tumor‐to‐whole‐egg uptake ratio proved robust as a normalized PET endpoint, compensating for inter‐individual variability in radiotracer injection efficiency inherent to the CAM model. Together, these data position the CAM‐[^18^F]FDG‐PET/CT platform as an intermediate‐throughput tool for quantitative functional assessment of drug efficacy in preclinical oncology research, with endpoints directly analogous to those used in clinical PET‐CT treatment monitoring.

### Overcoming the blood–brain barrier

In view of the limited drug penetration across the blood–brain barrier (BBB) in glioblastoma, we propose local postoperative intracavitary administration of the investigated drug combination via an Ommaya reservoir directly into the resection cavity, in order to bypass the BBB and achieve high intracerebral concentrations with reduced systemic exposure [78, 79]. The feasibility of drug release into the resection cavity is supported by current clinical data and recent reviews on local drug delivery [80, 81]. As a next step, intracranial mouse models are required to systematically assess distribution, pharmacodynamics, and potential neurotoxicity of direct CNS administration; the focus of the present study, however, was first to establish the molecular and functional efficacy of the drug combination. Since the intended route of administration is local intracavitary delivery rather than systemic therapy, systemic exposure, and hence the risk of extra‐cerebral toxicity affecting bone marrow, liver or kidney, is expected to remain minimal; a meaningful assessment of this question requires an intact organism and is therefore beyond the scope of the present study.

A direct comparison against the current standard of care, that is temozolomide plus radiotherapy, would be the clinically more familiar framing and is the decisive next step. Two considerations argue for the present design as an intermediate stage. First, temozolomide is already included as one of the four apoptosis‐inducing agents, so that the standard alkylating backbone is represented. Second, the present study establishes the rationale for additionally targeting the PAM pathway and identifies the more effective of the two clinically available inhibitors; defined dosing, a formal analysis of the synergistic interactions, data from orthotopic mouse models and a head‐to‐head comparison against temozolomide plus radiotherapy are the subsequent steps.

### Limitations of the study

Several limitations of this study should be stated explicitly. First, no data on extra‐cerebral toxicity were generated. Organotypic mouse brain slice cultures and the CAM model address the CNS compartment that is directly exposed under the intracavitary application proposed here, but adverse effects on bone marrow, liver, or kidney can only be assessed meaningfully in an intact organism; systemic tolerability will therefore be addressed in the future using orthotopic mouse models. Second, phosphorylation‐specific endpoints were not assessed, so that direct biochemical confirmation of the differential target engagement of Rapamycin and Dactolisib is still lacking and will be included in follow‐up studies. Third, the two commercial lines U87 and U251 are long‐established serum cultures for which published molecular subtype assignments are discordant, the ATCC U87 stock is a misidentified line whose donor is unknown, and U251 sublines are subject to genetic drift, so that the panel does not permit conclusions with respect to a defined molecular glioblastoma subtype.

Fourth, only a single patient‐derived stem‐like cell line was used, so that the inter‐patient heterogeneity of glioblastoma is not captured. Fifth, [^18^F]FDG rather than the clinically established amino‐acid tracer [^18^F]FET was used for PET imaging; this is appropriate in the CAM model, in which no adjacent brain parenchyma contributes background signal, but it limits direct comparability with clinical FET‐PET, and orthotopic models should be imaged with [^18^F]FET. Finally, the present study establishes the rationale of the combination but does not yet provide defined dosing, a formal analysis of the synergistic interactions, orthotopic *in vivo* data, or a comparison against temozolomide plus radiotherapy. Each of these points defines a next step rather than a qualification of the findings reported here.

## Conclusions

In summary, our findings support an integrated strategy that co‐targets glioblastoma cell survival programs and adaptive resistance, with Dactolisib, EVA, and chemotherapy outperforming Rapamycin combinations in reducing viability and activating apoptosis across glioblastoma cell lines and patient‐derived GSCs. [^18^F]FDG‐PET/CT imaging of CAM xenografts confirmed that these effects translate into measurable suppression of tumor glycolytic activity, providing a functional imaging correlate of therapeutic efficacy. Our combinatorial approach holds promise to improve outcomes by simultaneously disrupting multiple survival pathways while showing no concerning toxicity in mouse brain slices and the CAM model, thereby meriting further preclinical evaluation in intracranial models and subsequent clinical exploration.

## Conflicts of interest

The authors declare no conflict of interest.

## Author contributions

KZ, LM, AP, JPS, AHM, AN, AB, CN, JWB, and TR made substantial contributions to the conception and design of the work. Data were primarily acquired and analyzed by KZ, LM, MB, KO, CB, MP, BHY, PDF, HR, FH, JJ, JWB, and TR, with interpretation by KZ, LM, HU, MMCB, MWR, JPS, AHM, AN, AB, SB, BHY, PDF, ML, CN, JWB, and TR. KZ, LM, BHY, PDF, JWB, and TR drafted the manuscript and made substantial revisions. All authors read and approved the final manuscript.

## Supporting information


**Table S1.** Primary and secondary antibody information.
**Fig. S1**. Dose–response curves for 5‐Aza in U87 (A), U251 (B), and a patient‐derived GSC (C).
**Fig. S2**. Relative mRNA expression levels of mTOR, AKT2, PIK3CA, Bcl‐2, Mcl‐1, and XPO1 following treatment with A1210477, Venetoclax, Eltanexor, Rapamycin, Dactolisib, or EVA (Eltanexor, Venetoclax, A1210477) in combination with Rapamycin or Dactolisib.
**Fig. S3**. Protein levels of XPO1, Bcl‐2, and Mcl‐1 following combinatorial treatment with EVA and either Rapamycin or Dactolisib in U87 (A, B), U251 (C, D), and GSCs (E, F), assessed by western blotting.
**Fig. S4**. Protein level changes in mTOR, PIK3CA, and AKT2 after combinational treatment with Rapamycin or Dactolisib and EVA.
**Fig. S5**. Cell cycle distribution was analyzed by flow cytometry following PI staining.
**Fig. S6**. Cell viability was visualized by changes in the morphology after staining with DAPI.
**Fig. S7**. Autophagy‐related protein modifications following combined treatment with chemotherapeutics, EVA, and either Rapamycin or Dactolisib in U87 (A, B), U251 (C, D), and GSCs (E, F).

## Data Availability

The data that support the findings of this study are available on request from the corresponding author.
