# Supplementary material for: Dactolisib outperforms Rapamycin in potentiating apoptosis induced by XPO1, Bcl‐2 and Mcl‐1 blockade in preclinical glioblastoma models
Source: FEBS Open Bio. 2026 Oct 24:10.1002/2211-5463.70357. Online ahead of print. doi: 10.1002/2211-5463.70357 (PMC13695038; doi:10.1002/2211-5463.70357)
Supplement: Supplementary file 1 [file FEB4-9999-0-s001.zip › SUPPORTING_INFORMATION_FIGURES-edited.pdf]

SUPPLEMENTARY TABLES

Supplementary Table 1

Table S1: Primary and secondary antibody information

Antibody	Species	MV (kDa)	Article No	Company	Blocking Buffer	Dilution
AKT2	Rabbit	60	3063S	Cell Signaling	5% milk	1:1000 in 5% milk
Bcl-2	Mouse	25	MAB8272	R & D	5% milk	0.2μg/ml in 5% milk
LC3	Mouse	14,16	sc-271625	Santa Cruz Biotechnology	5% milk	1:500 in 5% milk
Mcl-1	Rabbit	35	94296S	Cell Signaling	5% milk	1:1000 in 5% BSA
mTOR	Rabbit	289	2983S	Cell Signaling	5% milk	1:1000 in 5% BSA
PIK3CA	Rabbit	110	4249S	Cell Signaling	5% milk	1:1000 in 5% BSA
p62	Rabbit	62	5114S	Cell Signaling	5% milk	1:1000 in 5% BSA
Tubulin	Rabbit	55	NB600-936	Novus Biologicals	5% milk	1:2000 in 5% milk
XPO1	Rabbit	123	46249S	Cell Signaling	5% milk	1:1000 in 5% BSA
Anti-Mouse	Donkey	-	ab97030	Abcam	-	1:4000 in 5% milk
Anti-Rabbit	Donkey	-	ab97064	Abcam	-	1:4000 in 5% milk or BSA

SUPPLEMENTARY FIGURES

Supplementary Figure 1

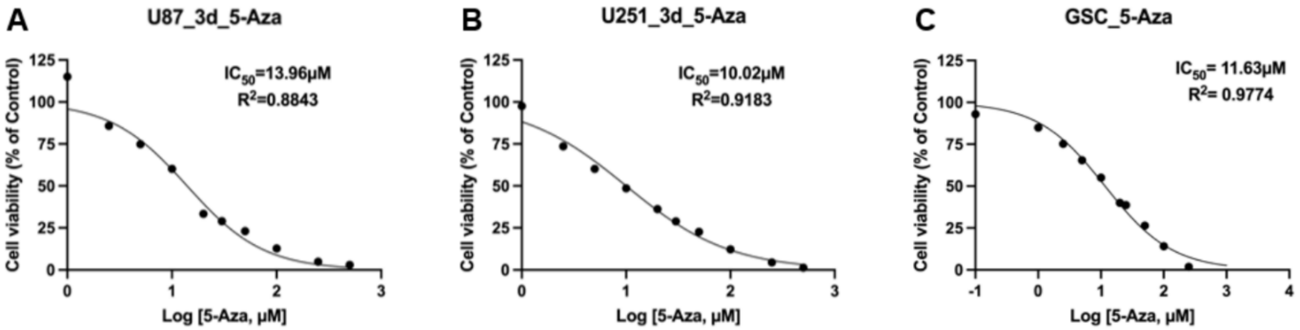

Figure S1: Dose–response curves for 5-Aza in U87 (A), U251 (B), and a patient-derived GSC (C). Generated from triplicate measurements across three independent experiments. Cell viability was assessed using CellTiter-Glo, and IC_{50} values were calculated by non-linear least-squares regression. IC_{50} values (µM) and corresponding regression coefficients are indicated in the respective plots.

Supplementary Figure 2

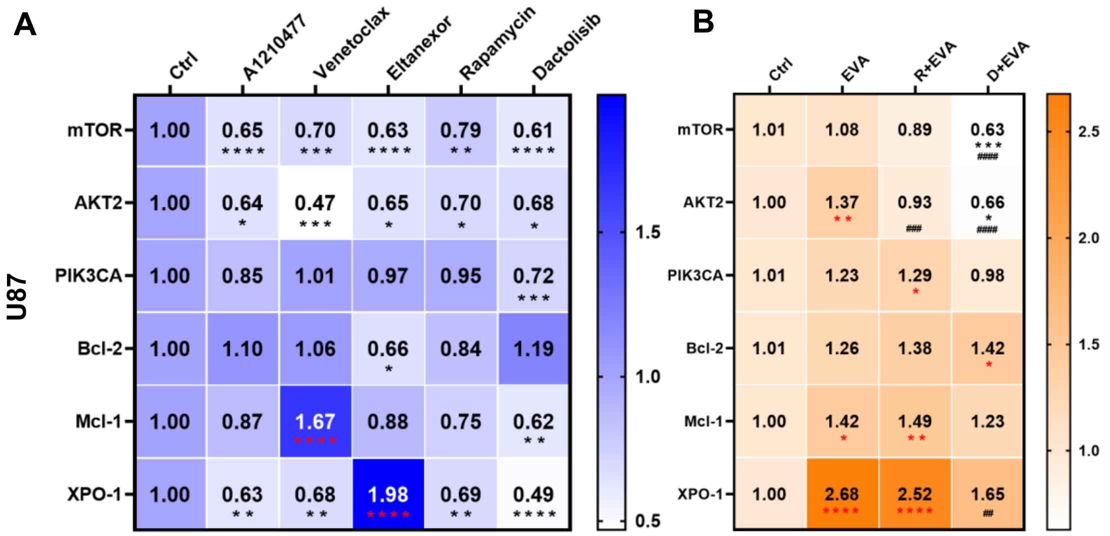

Figure S2: Relative mRNA expression levels of mTOR, AKT2, PIK3CA, Bcl-2, Mcl-1, and XPO1 following treatment with A1210477, Venetoclax, Eltanexor, Rapamycin, Dactolisib, or EVA (Eltanexor, Venetoclax, A1210477) in combination with Rapamycin or Dactolisib. U87 cells were treated for 24 h (A1210477: 1 µM; Venetoclax: 1 µM; Eltanexor: 100 nM; Rapamycin: 10 µM; Dactolisib: 250 nM). Red indicates upregulation, black indicates downregulation. Expression levels are based on mean values from three independent experiments relative to untreated controls. One-way ANOVA with post hoc (Tukey HSD) test was performed. * = vs. control, # = vs. EVA. */# $p < 0.05$, **/## $p < 0.01$, ***/### $p < 0.001$; ****/#### $p < 0.0001$; ns = not significant.

Supplementary Figure 3

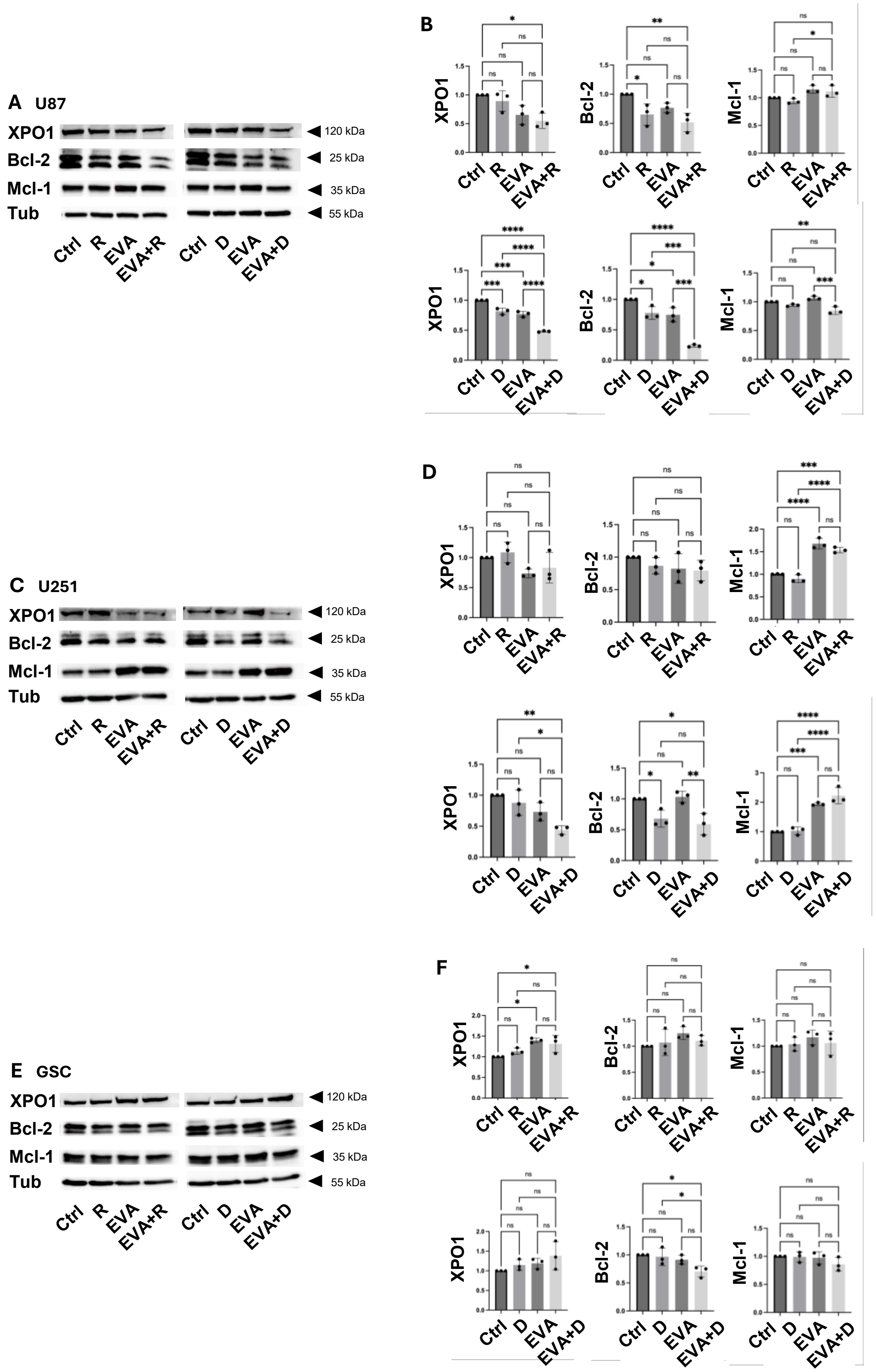

Figure S3: Protein levels of XPO1, Bcl-2, and Mcl-1 following combinatorial treatment with EVA and either Rapamycin or Dactolisib in U87 (A, B), U251 (C, D), and GSCs (E, F), assessed by Western blotting. U87 and U251 cells were treated for 24 h (A1210477: 1 µM; Venetoclax: 1 µM; Eltanexor: 100 nM; Rapamycin: 10 µM; Dactolisib: 250 nM), and GSCs for 48 h (A1210477: 100 nM; Venetoclax: 100 nM; Eltanexor: 10 nM; Rapamycin: 1 µM; Dactolisib: 100 nM). Quantification from three independent experiments is shown in panels B, D, and F. Data are presented as mean ± SD. Statistical significance was determined using one-way ANOVA followed by post hoc testing. *$p < 0.05$, **$p < 0.01$, ***$p < 0.001$, ****$p < 0.0001$, ns = not significant. The complete, uncropped membranes including molecular weight markers are provided in the supplementary material, in which the region shown in each panel is outlined.

Supplementary Figure 4

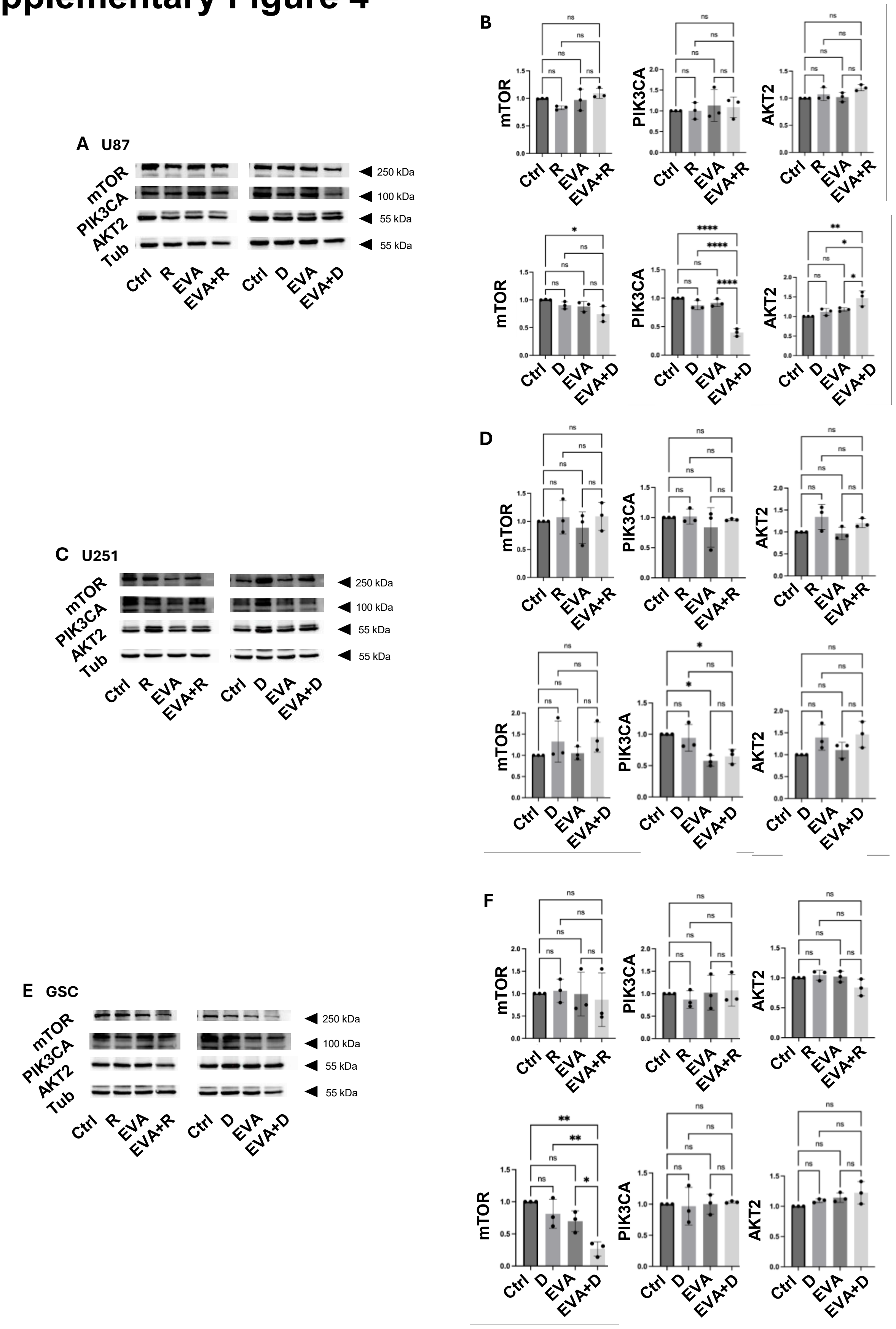

Figure S4: Protein level changes in mTOR, PIK3CA, and AKT2 after combinational treatment with Rapamycin or Dactolisib and EVA. Assessed via western blot in U87 **(A, B)**, and U251 **(C, D)**, and GSCs **(E, F)** with the same drug concentrations and incubation time as in Fig 3. Three independent experiments were performed for quantitative analysis **(B, D, F)**. Data are presented as mean ± SD. One-way ANOVA with a consecutive post-hoc test was used to analyze, * $p < 0.05$, ** $p < 0.01$, *** $p < 0.001$, **** $p < 0.0001$, ns: no significant. The complete, uncropped membranes including molecular weight markers are provided in the supplementary material, in which the region shown in each panel is outlined.

Supplementary Figure 5

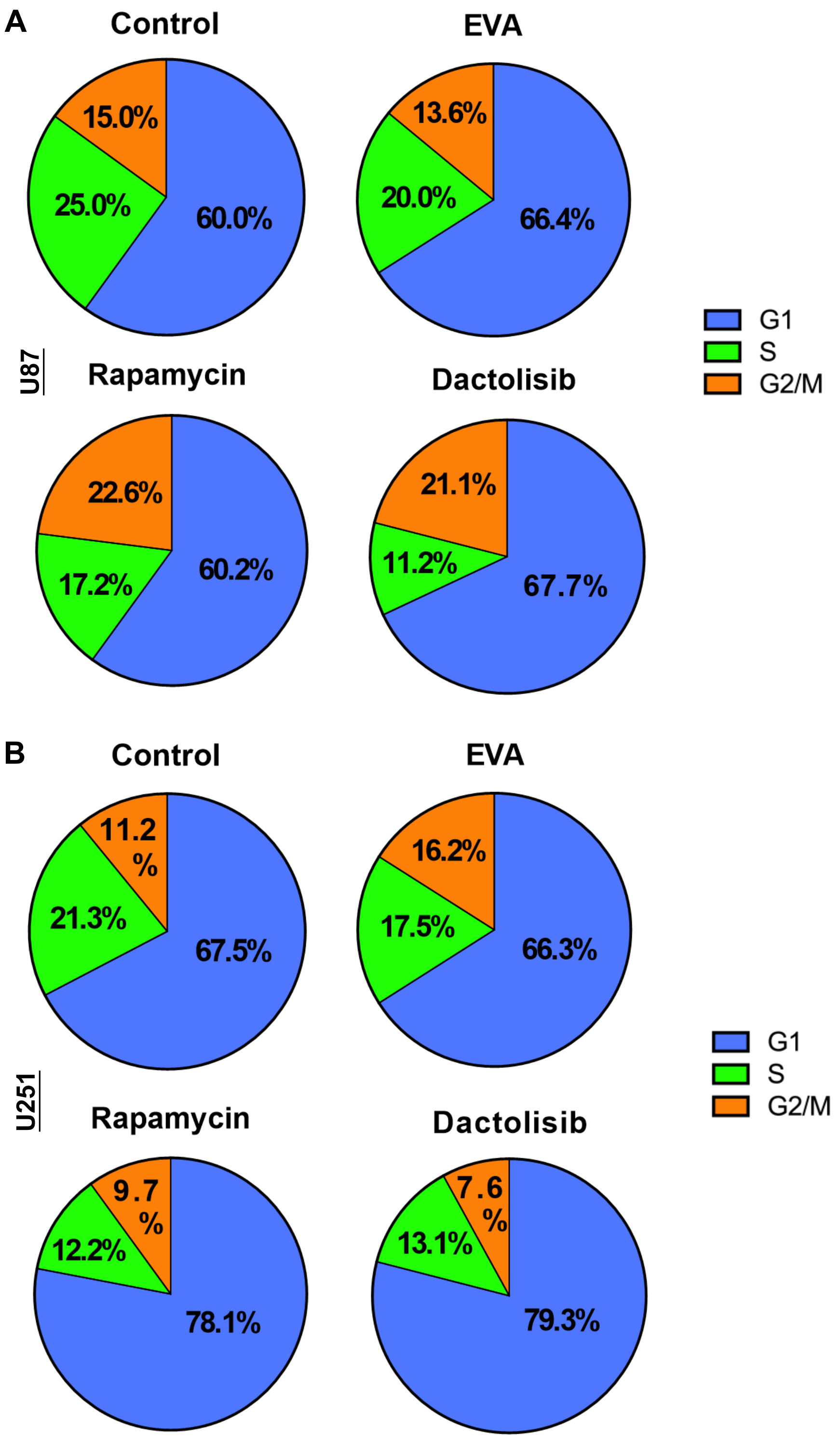

Figure S5: Cell cycle distribution was analyzed by flow cytometry following PI-staining. U87 and U251 cells were treated for 24 h (A1210477: 1 µM; Venetoclax: 1 µM; Eltanexor: 100 nM; Rapamycin: 10 µM; Dactolisib: 250 nM). Pie charts illustrate the proportions of cells in G1 (blue), S (green), and G2/M (orange) phases. Representative experiments of three independent experiments are shown; the mean ± SD of all three experiments are given in section 3.6.

Supplementary Figure 6

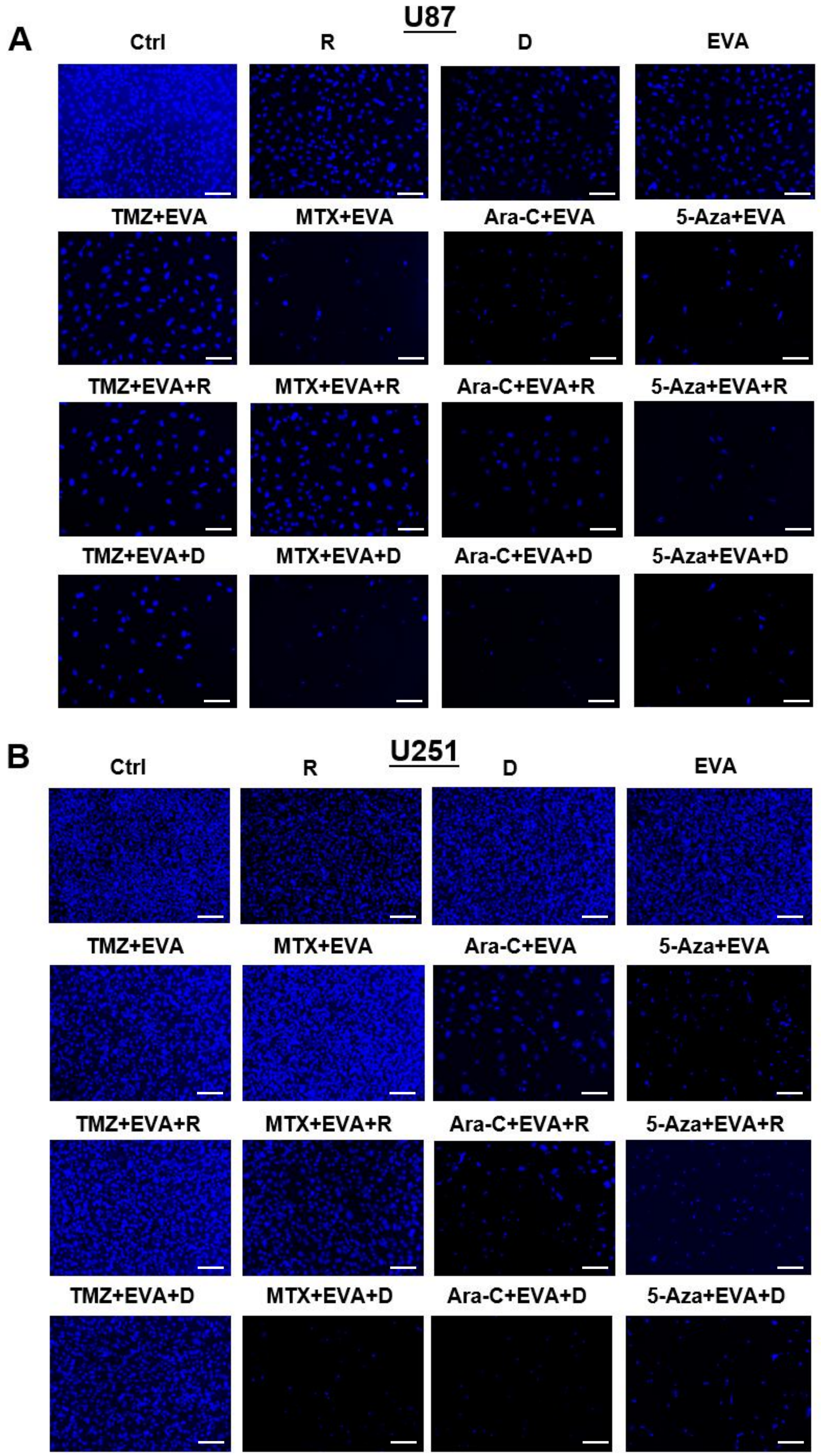

Figure S6: Cell viability was visualized by changes in the morphology after staining with DAPI. U87 **(A)** and U251 **(B)** cells were treated for 72 h, GSCs were treated for 10 days. Sublethal doses of Venetoclax, A1210477 (1 µM each) and Eltanexor (100 nM) were used in U87 and U251 cells as well as in GSCs (A 100 nM, V 100 nM, E 10 nM) in combination with chemotherapeutics (same concentration as in Fig. 4) to evaluate synergistic effects. Scale bars indicate 100 µm.

Supplementary Figure 7

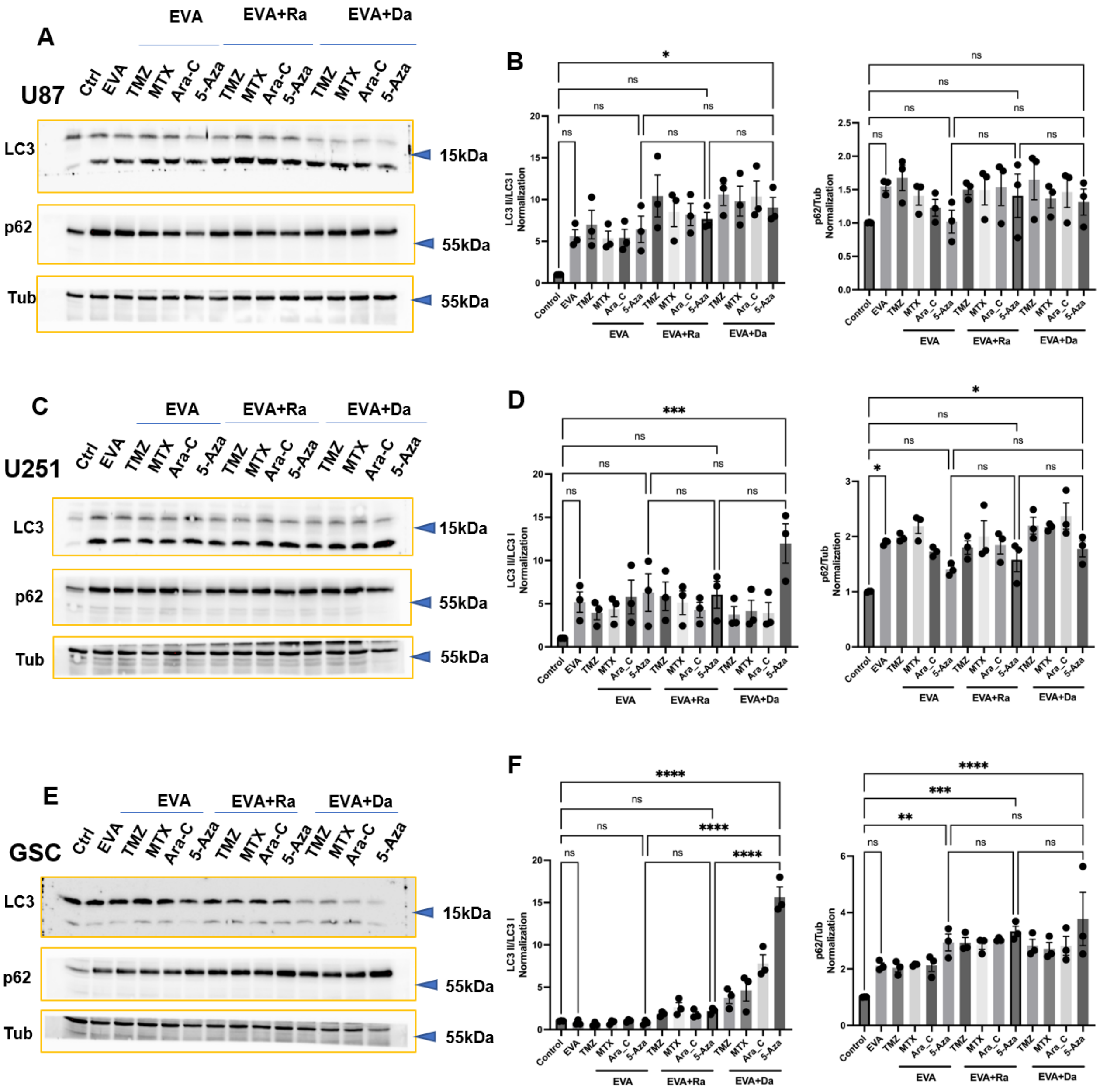

Figure S7: Autophagy-related protein modifications following combined treatment with chemotherapeutics, EVA, and either Rapamycin or Dactolisib in U87 (A, B), U251 (C, D), and GSCs (E, F). Cells were treated for 24 h with the same drug concentrations as in Fig. 4, and autophagy-related proteins were assessed by Western blot. Quantification from three independent experiments is shown in panels B, D, and F. Data are presented as mean ± SEM. One-way ANOVA with a consecutive post-hoc test was used to analyze, * $p < 0.05$, ** $p < 0.01$, *** $p < 0.001$, **** $p < 0.0001$, ns: no significant.
